# DNA Priming for Seasonal Influenza Vaccine: A Phase 1b Double-Blind Randomized Clinical Trial

**DOI:** 10.1371/journal.pone.0125914

**Published:** 2015-05-07

**Authors:** Julie E. Ledgerwood, Abbie R. Bellamy, Robert Belshe, David I. Bernstein, Srilatha Edupuganti, Shital M. Patel, Phyllis Renehan, Thad Zajdowicz, Richard Schwartz, Richard Koup, Robert T. Bailer, Galina V. Yamshchikov, Mary E. Enama, Uzma Sarwar, Brenda Larkin, Barney S. Graham, VRC 701 study team

**Affiliations:** 1 Vaccine Research Center, National Institutes of Allergy and Infectious Diseases, National Institutes of Health, Bethesda, Maryland, United States of America; 2 The EMMES Corporation, Rockville, Maryland, United States of America; 3 Edward A. Daisy Research Center, Saint Louis University, Saint Louis, Missouri, United States of America; 4 Cincinnati Children’s Hospital Medical Center, University of Cincinnati, Cincinnati, Ohio, United States of America; 5 Department of Medicine, Division of Infectious Diseases, Emory University School of Medicine, Atlanta, Georgia, United States of America; 6 Department of Medicine and Molecular Virology and Microbiology, Baylor College of Medicine Houston, Texas, United States of America; Public Health England, UNITED KINGDOM

## Abstract

**Background:**

The efficacy of current influenza vaccines is limited in vulnerable populations. DNA vaccines can be produced rapidly, and may offer a potential strategy to improve vaccine immunogenicity, indicated by studies with H5 influenza DNA vaccine prime followed by inactivated vaccine boost.

**Methods:**

Four sites enrolled healthy adults, randomized to receive 2011/12 seasonal influenza DNA vaccine prime (n=65) or phosphate buffered saline (PBS) (n=66) administered intramuscularly with Biojector. All subjects received the 2012/13 seasonal inactivated influenza vaccine, trivalent (IIV3) 36 weeks after the priming injection. Vaccine safety and tolerability was the primary objective and measurement of antibody response by hemagglutination inhibition (HAI) was the secondary objective.

**Results:**

The DNA vaccine prime-IIV3 boost regimen was safe and well tolerated. Significant differences in HAI responses between the DNA vaccine prime and the PBS prime groups were not detected in this study.

**Conclusion:**

While DNA priming significantly improved the response to a conventional monovalent H5 vaccine in a previous study, it was not effective in adults using seasonal influenza strains, possibly due to pre-existing immunity to the prime, unmatched prime and boost antigens, or the lengthy 36 week boost interval. Careful optimization of the DNA prime-IIV3 boost regimen as related to antigen matching, interval between vaccinations, and pre-existing immune responses to influenza is likely to be needed in further evaluations of this vaccine strategy. In particular, testing this concept in younger age groups with less prior exposure to seasonal influenza strains may be informative.

**Trial Registration:**

ClinicalTrials.gov NCT01498718

## Introduction

The first influenza vaccine was licensed in the US in the 1940s. In the decades since, the accumulated data support the continued use of vaccine to reduce community transmission and severity of influenza disease [1]. Annually, the World Health Organization (WHO), the U.S. FDA, and other advisory agencies make recommendations on the composition of the seasonal influenza vaccine; the FDA selects the strains to include in vaccines for the U.S. population. Recommendations for the Northern Hemisphere and for the Southern Hemisphere are considered at different times based on epidemiology data. Until recently, the annually licensed trivalent inactivated influenza vaccines (IIV3) consisted of 3 strains: influenza A (H1N1), influenza A (H3N2), and an influenza B virus. Beginning with the 2013–14 vaccines, quadrivalent influenza vaccines containing an additional influenza B virus strain were approved. Inactivated influenza vaccine manufacturing is labor-intensive and rapid adjustment in production capacity in response to emerging epidemics/pandemics is limited by the availability of egg-adapted strains, as well as the egg supply needed for production. In addition to the need for more flexible and scalable manufacturing, there is also a need for improved levels of efficacy in vulnerable populations such as the elderly, young children, pregnant women and the immunocompromised. DNA vaccines can be manufactured rapidly because the sequences for novel strains can be incorporated quickly and the manufacturing process is efficient [2]. Induction of both humoral and cellular immunity by DNA vaccines used alone or in a prime-boost regimen may offer broader immune response and protection as it has been demonstrated in animal studies [3–6].

The DNA vaccine prime-inactivated vaccine boost strategy evaluated in the current study has been shown to improve the immune response for an H5N1 influenza strain [7, 8]. Based on experience with H5 influenza DNA vaccine priming, we initiated a series of studies with seasonal influenza DNA vaccine prime followed by IIV3 boost to assess the generalizability of the H5N1 findings. DNA priming may be a useful strategy for the older adult and pediatric populations for which IIV3 alone has lower efficacy. In the previous clinical studies of H5 DNA prime-H5N1 monovalent inactivated vaccine (MIV) boost, it was found that antibody responses are 4–6 fold higher after the boost when the prime-boost interval is 3–6 months compared to a shorter interval [7, 8]. To evaluate a prime-boost interval across two influenza seasons, in the VRC 701 clinical trial described here, DNA priming followed by a IIV3 boost 36 weeks later was compared to IIV3 alone.

## Methods

The protocol for this trial and supporting CONSORT checklist are available as supporting information; see S1 Protocol and S1 CONSORT Checklist.

### Ethics Statement

The study was approved by the IRBs at Saint Louis University, Cincinnati Children’s Hospital Medical Center Cincinnati, Emory University, and Baylor College of Medicine. All subjects completed the consent process and signed written informed consent documents.

The study was conducted following guidelines for conducting clinical research with human subjects from the US Department of Health and Human Services, and was performed in accordance with 45 CFR Part 46, U.S. Food and Drug Administration regulations for investigational products, and principles expressed in the Declaration of Helsinki.

### Study Design

VRC 701 was a phase 1b double-blinded, randomized, controlled trial to assess the safety and immunogenicity of a 2011/12 seasonal DNA vaccine prime followed by a licensed 2012/13 IIV3 boost 36 weeks later. The comparator group received phosphate buffered saline (PBS) prime and the 2012/13 seasonal IIV3 boost on the same schedule. Healthy adults, aged 18–70 years, who had received the 2011/12 licensed IIV3 at 8 or more weeks prior to enrollment, were eligible to participate in the trial. Subjects who were pregnant or breastfeeding; had recent receipt of immune modulating medical products; had contraindication to receiving influenza vaccine or history of serious vaccine reactions, were not eligible for participation.

Subjects were randomized to the DNA-IIV3 or PBS-IIV3 groups with equal allocation stratified within each age stratum (18–50 years or 51–70 years) and site. The randomization sequence was generated by the trial statistician in SAS using permuted blocked randomization with randomly selected block sizes of two or four. Upon enrollment, each subject was assigned a randomization number from the electronic data entry system that corresponded to a treatment on a randomization list available to the unblinded vaccine administrator. All prime injections, whether DNA vaccine or PBS, were administered in a blinded manner intramuscularly in the deltoid with the Biojector device (Bioject; Tualatin, Oregon, USA) on the day of enrollment (Day 0). The 2012/13 seasonal IIV3 boost (Fluzone, 45 mcg) was administered at study week 36. Subjects, clinical site staff, and laboratory staff were blinded throughout the duration of the trial.

The subjects completed diary cards to assess solicited local and systemic reactogenicity for 7 days after each vaccination; all adverse events that occurred within 28 days of either vaccination, and all SAEs or influenza-like illnesses throughout the 60 week duration of the trial were recorded. Adverse events were coded using the Medical Dictionary for Regulatory Activities (MedDRA). Blood samples collected prior to each vaccination and 4 weeks after the boost vaccination were tested for immune response to the 5 influenza strains included in the 2011/12 and/or 2012/13 seasonal influenza vaccines (Table 1).

**Table 1:** Influenza strains included in DNA vaccine prime and IIV3 boost.

*DNA vaccine prime*	*IIV3 boost*	*Strains for HAI testing*
A/California/04/2009(H1N1)	A/California/07/2009(H1N1)	A/California/07/2009(H1N1)
A/Perth/16/2009(H3N2)		A/Perth/16/2009(H3N2)
	A/Victoria/361/2011(H3N2)	A/Victoria/361/2011(H3N2)
B/Brisbane/60/2008		B/Brisbane/60/2008
	B/Wisconsin/1/2010-like: B/Texas/6/2011	B/Wisconsin/1/2010

The trial was conducted at 4 clinical sites in the United States: Center for Vaccine Development, Saint Louis University, Saint Louis, Missouri; Cincinnati Children’s Hospital Medical Center Cincinnati, Ohio; Hope Clinic of the Emory Vaccine Center, Atlanta, Georgia; and Baylor College of Medicine, Houston, Texas. The first subject was screened for recruitment on December 20, 2011, study vaccinations began on January 10, 2012 and study follow-up continued through April 17, 2013.

### Vaccines

The investigational trivalent 2011/12 seasonal influenza DNA vaccine (VRC-FLUDNA061-00-VP) consisted of three closed-circular plasmid DNA macromolecules (VRC-9328, VRC-2439 and VRC-9323), in equal amounts by weight, that express influenza hemagglutinin (HA) sequences for strains that match the 2011/12 licensed IIV3: A/California/04/2009(H1N1), Gene Bank accession #GQ117044; A/Perth/16/2009(H3N2), accession #ACS71642; and B/Brisbane/60/2008, accession #ACN29380, under control of the CMV/R promoter. The plasmid DNA was manufactured at the VRC/NIAID/Vaccine Pilot Plant operated by Leidos Biomedical Research, Incorporated (formerly Science Applications International Corporation, (SAIC), Frederick, Maryland) under current Good Manufacturing Practices at 4 mg/mL in phosphate buffered saline (PBS).

The licensed IIV3 for the booster dose, Fluzone (Sanofi Pasteur, Inc., Swiftwater, Pennsylvania, USA), contained the 3 influenza strains approved for the 2012/13 seasonal IIV3: A/California/7/2009(H1N1), A/Victoria/361/2011(H3N2), B/Wisconsin/1/2010–like: B/Texas/6/2011.

### Immunogenicity Assay Methods

Hemagglutinin (HA)-specific antibody as measured by Hemagglutination Inhibition (HAI) assay is the traditional benchmark measure of immune response to influenza vaccines. The primary immunogenicity time point was 4 weeks after the boost. The measurement of antibody by HAI assay was performed by a validated laboratory method at Bioqual, Inc., 9600 Medical Center Drive, Rockville, Maryland 20850. Briefly, the HAI assays were done in V-bottom 96-well plates using four hemagglutinating units of virus and 0.5% turkey red blood cells.

### Statistical Analysis

The primary objective of the study was to assess the safety and tolerability of the DNA prime-IIV3 boost regimen. The secondary objective was to assess the frequency of positive responders by HAI at four weeks after the IIV3 boost, defined per the FDA criteria for seroconversion relative to baseline as either a baseline (Day 0) HAI titer < 1:10 and a post boost HAI titer ≥ 1:40 or a baseline HAI titer ≥ 1:10 and a minimum four-fold rise from baseline [9]. As an exploratory objective we summarized the proportion of subjects with seroconversion relative to their pre-boost titer defined as either a pre-boost (week 36) HAI titer < 1:10 and a post boost HAI titer ≥ 1:40 or a pre-boost HAI titer ≥ 1:10 and a minimum four-fold rise post-boost. Magnitude of immune response was estimated as the HAI geometric mean titer (GMT). Comparisons were made between the DNA-prime and control groups overall, and within each age stratum, using Fisher’s exact test for proportions of responders and t-test for response magnitude using log-transformed HAI titers. Statisical significance was considered at a level of alpha = 0.05 without adjustment for multiple comparisons. As an exploratory post-hoc analysis, a linear regression model was fit to explore the effect of age stratum (18–50, 51–70) on log-transformed HAI titers when adjusting for treatment group. Statistical analyses were performed in SAS 9.3 (SAS Institute, Cary, North Carolina).

## Results

### Study Population

**Fig 1:**
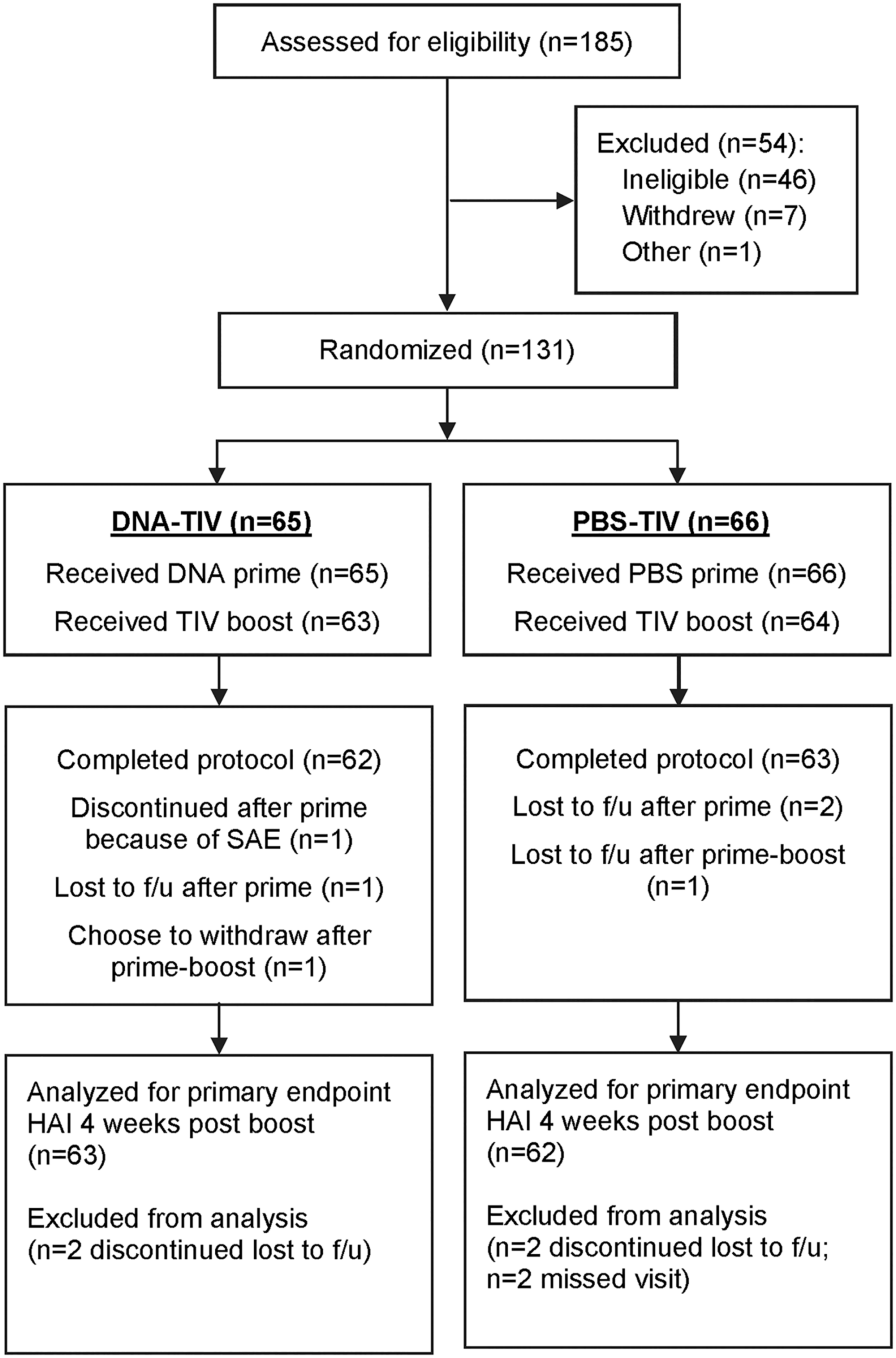
VRC 701 Consort diagram of subject disposition.

185 volunteers were screened at 4 sites, and 131 eligible healthy adults were enrolled in the trial between 12/9/2011 and 2/6/2012. All 131 healthy adults who were enrolled (Fig 1) received the prime vaccination (65 DNA, 66 PBS Control), 127 received the boost vaccination, and 125 subjects completed the study. Subject demographics are shown in Table 2. The majority of the study population was female (71%), white (85%) and non-Hispanic (95%). The average age was 46.8 years (range 20 to 70 years); 66 subjects in the 18–50 year old stratum and 65 subjects in the 51–70 year old stratum. The average body mass index (BMI) was 27.1 (range 18.5 to 39.6). Previous influenza vaccination history is summarized by treatment group and by age strata in Table 3.

**Table 2:** Subject Demographics.

		VRC 701 DNA-IIV3 (N = 65)	VRC 701 PBS-IIV3 (N = 66)	VRC 701 All Subjects (N = 131)
**GENDER**	Male	20 (30.8%)	18 (27.3%)	38 (29.0%)
	Female	45 (69.2%)	48 (72.7%)	93 (71.0%)
**AGE**	18–50	32 (49.2%)	34 (51.5%)	66 (50.4%)
	51–70	33 (50.8%)	32 (48.5%)	65 (49.6%)
	Mean (S.D.)	47.1 (15)	46.4 (14)	46.8 (14)
	Range	[20, 70]	[22, 70]	[20, 70]
**RACE**	Asian	1 (1.5%)	1 (1.5%)	2 (1.5%)
	Black or African American	12 (18.5%)	4 (6.1%)	16 (12.2%)
	White	52 (80.0%)	60 (90.9%)	112 (85.5%)
	Multiracial	0 (0.0%)	1 (1.5%)	1 (0.8%)
**ETHNICITY**	Non-Hispanic/Latino	60 (92.3%)	64 (97.0%)	124 (94.7%)
	Hispanic/Latino	5 (7.7%)	2 (3.0%)	7 (5.3%)
**BMI**				
	Mean (S.D.)	27.7 (5.2)	26.5 (5.1)	27.1 (5.2)
	Range	[18.6, 39.6]	[18.5, 38.5]	[18.5, 39.6]

**Table 3:** Frequency of previous seasonal influenza vaccinations.

*Number of influenza vaccinations in the last 5 years* *	*DNA-IIV3 (n = 65)*	*PBS-IIV3 (n = 66)*	*Age 18–50 years (n = 66)*	*Age 51–70 years (n = 65)*	*Overall (n = 131)*
>5 times	16 (25%)	10 (15%)	11 (17%)	15 (23%)	26 (20%)
3–5 times	41 (63%)	49 (74%)	41 (62%)	49 (75%)	90 (69%)
1–2 times	8 (12%)	7 (11%)	14 (21%)	1 (2%)	15 (11%)

*All subjects received 2011/2012 IIV3 at least 8 weeks prior to enrollment in the trial.

### Vaccine Reactogenicity and Safety

The investigational DNA vaccine was assessed as safe and well tolerated. A total of 6 SAEs were reported (3 in DNA-IIV3 group and 3 in PBS-IIV3 group) from the time of prime vaccination through the 60 weeks of follow-up; none were assessed as related to study injections. There were 4 unsolicited adverse events, injection site rash (1 day post vaccination), injection site scab (2 events; 3 and 5 days post vaccination), and injection site pruritus (2 days post vaccination), considered definitely related to DNA vaccination; all were mild and resolved without sequelae. The most commonly reported AEs within 28 days following the prime vaccination were: upper respiratory tract infection (4.6% DNA; 9.1% PBS), bruising at the injection site (6.2% DNA; 6.1% PBS) and influenza like illness (4.6% DNA; 7.6% PBS). 18 cases of influenza-like illnesses were reported by 17 subjects: 7 cases in the 2011/12 influenza season and 11 cases in the 2012/2013 influenza season; 8 cases in the DNA-IIV3 group and 9 cases (one subject reported 2 cases) in the PBS-IIV3 treatment group.

No severe solicited injection site or systemic reactions were reported within 7 days of injection (Table 4). For the prime vaccination, the most common injection site reaction was mild or moderate pain/tenderness, reported more frequently by subjects who received the DNA vaccine (71%, 95%CI: 58–81%) compared with the PBS control (39%, 95%CI: 27–52%). Pain/tenderness was also the most common injection site symptom following the IIV3 boost, but with no difference between subjects primed with DNA (41%, 95%CI: 29–54%) compared with the placebo primed controls (45%, 95%CI: 32–58%). The investigational DNA vaccine was associated with an increased frequency of local pain/tenderness compared to PBS control or IIV3 (p <0.001 for both comparisons). Overall, more subjects reported local reactogenicity following the DNA prime (75%, 95%CI: 63–85%) compared with those who received PBS control (42%, 95%CI: 30–55%; p = 0.0001), though most symptoms were mild.

**Table 4:** Solicited Reactogencity within 7 Days of Injection.

	Vaccination 1—Prime Injection		Vaccination 2—IIV3 Boost		
SymptomsIntensity	DNA Vaccine (N = 65)	PBS Injection (N = 66)	Following DNA Prime (N = 63)	Following PBS Prime (N = 64)	All (N = 127)
***Local Reactogenicity*, *subjects (%)***					
**PAIN/TENDERNESS**					
None	19(29.2)	40(60.6)	37(58.7)	35(54.7)	72(56.7)
Mild	42(64.6)	26(39.4)	24(38.1)	27(42.2)	51(40.2)
Moderate	4(6.2)	0(0.0)	2(3.2)	2(3.1)	4(3.1)
**SWELLING**					
None	60(92.3)	63(95.5)	63(100.0)	64(100.0)	127(100.0)
Mild	4(6.2)	2(3.0)	0(0.0)	0(0.0)	0(0.0)
Moderate	1(1.5)	1(1.5)	0(0.0)	0(0.0)	0(0.0)
**REDNESS**					
None	62(95.4)	64(97.0)	62(98.4)	64(100.0)	126(99.2)
Mild	3(4.6)	2(3.0)	1(1.6)	0(0.0)	1(0.8)
Moderate	0(0.0)	0(0.0)	0(0.0)	0(0.0)	0(0.0)
**ANY LOCAL SYMPTOM**					
None	16(24.6)	38(57.6)	37(58.7)	35(54.7)	72(56.7)
Mild	44(67.7)	27(40.9)	24(38.1)	27(42.2)	51(40.2)
Moderate	5(7.7)	1(1.5)	2(3.2)	2(3.1)	4(3.1)
***Systemic Reactogenicity*, *subjects (%)***					
**MALAISE**					
None	57(87.7)	58(87.9)	56(88.9)	56(87.5)	112(88.2)
Mild	6(9.2)	6(9.1)	7(11.1)	6(9.4)	13(10.2)
Moderate	2(3.1)	2(3.0)	0(0.0)	2(3.1)	2(1.6)
**MYALGIA**					
None	58(89.2)	63(95.5)	60(95.2)	60(93.8)	120(94.5)
Mild	7(10.8)	2(3.0)	3(4.8)	3(4.7)	6(4.7)
Moderate	0(0.0)	1(1.5)	0(0.0)	1(1.6)	1(0.8)
**HEADACHE**					
None	52(80.0)	53(80.3)	55(87.3)	55(85.9)	110(86.6)
Mild	11(16.9)	11(16.7)	8(12.7)	5(7.8)	13(10.2)
Moderate	2(3.1)	2(3.0)	0(0.0)	4(6.3)	4(3.1)
**CHILLS**					
None	61(93.8)	63(95.5)	63(100.0)	63(98.4)	126(99.2)
Mild	3(4.6)	2(3.0)	0(0.0)	0(0.0)	0(0.0)
Moderate	1(1.5)	1(1.5)	0(0.0)	1(1.6)	1(0.8)
**NAUSEA**					
None	62(95.4)	62(93.9)	62(98.4)	58(90.6)	120(94.5)
Mild	3(4.6)	3(4.5)	1(1.6)	6(9.4)	7(5.5)
Moderate	0(0.0)	1(1.5)	0(0.0)	0(0.0)	0(0.0)
**TEMPERATURE**					
None	65(100.0)	65(98.5)	63(100.0)	64(100.0)	127(100.0)
Mild	0(0.0)	1(1.5)	0(0.0)	0(0.0)	0(0.0)
Moderate	0(0.0)	0(0.0)	0(0.0)	0(0.0)	0(0.0)
**ANY SYSTEMIC SYMPTOM**					
None	47(72.3)	52(78.8)	52(82.5)	48(75.0)	100(78.7)
Mild	15(23.1)	12(18.2)	11(17.5)	12(18.8)	23(18.1)
Moderate	3(4.6)	2(3.0)	0(0.0)	4(6.3)	4(3.1)

Note: Subjects are counted once at the maximum severity reported for each symptom.

The proportion of subjects reporting systemic reactogenicity following the prime injection was similar for DNA prime (28%, 95%CI: 17–40%) compared with those who received PBS control (21%, 95%CI: 12–33%; p = 0.42). (Table 4). The most frequent systemic symptom post prime was headache, reported at equal frequency in both groups (20%, 95%CI: 11–31%). Although myalgia appeared to be more frequent among DNA vaccine recipients (11%, 95% CI: 4–21%, post DNA (all mild); 5% post PBS (95% CI: 1–13%, 3% mild, 2% moderate), the difference in frequency is not statistically significant (p = 0.20). Regardless of prime vaccination, systemic symptoms were reported at similar rates following the IIV3 boost (DNA-IIV3 18%, 95%CI: 9–29% and PBS-IIV3 18%, 95%CI: 15–37%).

### Immune Response

Administration of priming injections began in January 2012, and as a criterion for inclusion in the trial, all study participants received the 2011/12 IIV3 at least 8 weeks prior to priming injection. The pre-existing immunity was similar between the two study groups; 73% of DNA vaccine subjects and 83% of PBS control subjects had titers ≥ 1:40 as measured by HAI for A/California/07/2009 (H1N1), an antigen included in the DNA prime and IIV3 boost (Table 5).

**Table 5:** Rates of Positive Immune Response 4 Weeks post IIV3 boost as measured by HAI: % of subjects (95% CI).

Antigen	Immune Response Measure	*DNA-IIV3 (n = 65)*	*PBS-IIV3 (n = 66)*
***A/California/07/2009(H1N1) Represented in DNA Vaccine Prime and IIV3 Boost***	≥1:10 at baseline	94 (85–98)	97 (89–100)
	≥1:40 at baseline	83 (72–91)	73 (60–83)
	≥1:10 pre-boost	86 (75–93)	86 (75–93)
	≥1:40 pre-boost	71 (59–82)	63 (50–74)
	seroconversion relative to baseline	16 (8–27)	23 (13–36)
	seroconversion relative to pre-boost	30 (19–43)	37 (19–43)
***A/Perth/16/2009(H3N2) Represented in DNA Vaccine Prime***	≥1:10 at baseline	89 (79–96)	82 (70–90)
	≥1:40 at baseline	63 (50–75)	55 (42–67)
	≥1:10 pre-boost	81 (69–90)	69 (56–80)
	≥1:40 pre-boost	54 (41–67)	44 (31–47)
	seroconversion relative to baseline	22 (13–35)	26 (16–39)
	seroconversion relative to pre-boost	32 (20–45)	36 (24–49)
***B/Brisbane/60/2008 Represented in DNA Vaccine Prime***	≥1:10 at baseline	88 (77–95)	80 (69–90)
	≥1:40 at baseline	42 (29–54)	32 (21–44)
	≥1:10 pre-boost	70 (57–80)	64 (51–76)
	≥1:40 pre-boost	30 (19–43)	30 (19–42)
	seroconversion relative to baseline	8 (3–17)	8 (3–18)
	seroconversion relative to pre-boost	6 (2–16)	8 (3–18)
***A/Victoria/361/2011(H3N2) Represented in IIV3 Boost***	≥1:10 at baseline	100 (95–100)	99 (92–100)
	≥1:40 at baseline	86 (75–93)	77 (65–87)
	≥1:10 pre-boost	92 (82–97)	97 (90–100)
	≥1:40 pre-boost	76 (64–86)	70 (58–81)
	seroconversion relative to baseline	22 (13–36)	24 (14–37)
	seroconversion relative to pre-boost	30 (19–43)	44 (31–57)
***B/Wisconsin/1/2010 Represented in IIV3 Boost***	≥1:10 at baseline	43 (30–56)	41 (29–54)
	≥1:40 at baseline	18 (10–30)	18 (10–30)
	≥1:10 at pre-boost	46 (33–59)	36 (24–49)
	≥1:40 pre-boost	16 (8–27)	22 (13–34)
	seroconversion relative to baseline	22 (13–35)	27 (17–40)
	seroconversion relative to pre-boost	27 (17–40)	27 (17–40)

Seroconversion defined as four-fold increase in titer 4 weeks post boost if reference titer (baseline or pre-boost) is ≥1:10, or ≥1:40 if reference titer is < 1:10.

Baseline immune responses were similarly high for the other 2 influenza A strains, A/Perth/16/2009 (H3N2) and A/Victoria/361/2011 (H3N2) included in the DNA prime or IIV3 boost, respectively, and to B/Brisbane/60/2008 (DNA prime). Relatively lower frequency of pre-existing immune responses was detected to the influenza B strain, new for the 2012/13 season, B/Wisconsin/1/2010: 18% of subjects in both groups had a titer ≥1:40. The respective frequencies measured 36 weeks later, before the IIV3 boost, were similar to frequencies measured at baseline.

At baseline the magnitude of pre-existing immunity, GMT (95%CI), evaluated for all 131 subjects, was higher for influenza A strains [A/California/07/2009 (H1N1): 95(75–122), A/Perth/16/2009 (H3N2): 41 (33–53), A/Victoria/361/2011 (H3N2): 107 (86–135)] than B strains [B/Brisbane/60/2008: 23 (19–27); B/Wisconsin/1/2010: 10 (9–12)]. The highest baseline antibody among the influenza A strains was for A/Victoria/361/2011 (H3N2), which was the next 2012/13 seasonal strain and apparently had been circulating in the population for a period of time.

Subjects ages 18–50 years had a higher magnitude of pre-existing immunity compared to subjects age 51–70 years for 3 of 5 strains: A/California/07/2009 (H1N1) [18–50: 148 (105–207), 51–70: 61 (45–85); p = 0.0002], B/Brisbane/60/2008 [18–50: 28 (22–37), 51–70: 18 (15–23); p = 0.01], and B/Wisconsin/1/2010 [18–50: 13 (10–16), 51–70: 8 (7–10); p = 0.01]. Within each age stratum the GMTs were similar between the two treatment groups (Table 6).

**Table 6:** GMT for study groups as measured by HAI: GMT (95% CI).

Antigen	Time Point	All Subjects (n = 131)		18–50 years (n = 66)		51–70 years (n = 65)	
		DNA-IIV3 (n = 65)	PBS-IIV3 (n = 66)	DNA-IIV3 (n = 32)	PBS-IIV3 (n = 34)	DNA-IIV3 (n = 33)	PBS-IIV3 (n = 32)
***A/California/04/2009 (H1N1)*** *Represented in DNA Vaccine Prime and IIV3 Boost*	baseline	103.7 (72.8,147.6)	88.5 (63.1,124.2)	186.1 (118.8,291.4)	119.4 (71.7,199.0)	58.8 (36.1,95.8)	64.4 (41.4,100.2)
	pre-boost	67.6 (45.9,99.5)	55.1 (38.3,79.3)	124.1 (73.9,208.4)	86.6 (52.7,142.1)	37.5 (22.3,62.9)	35.1 (21.1,58.5)
	4 weeks post boost	159.3 (120.5,210.7)	158.7 (112.1,224.8)	281.6 (204.9,387.2)	209.4 (124.6,351.9)	91.7 (62.9,133.8)	122.4 (76.1,197.0)
***A/Perth/16/2009 (H3N2)*** *Represented in DNA Vaccine Prime*	baseline	47.1 (33.8,65.7)	36.9 (25.8,52.9)	55.7 (32.9,94.5)	42.8 (24.2,75.7)	40.0 (26.0,61.5)	31.5 (20.0,49.7)
	pre-boost	33.3 (23.8,46.5)	25.7 (17.6,37.5)	40.3 (24.4,66.4)	29.5 (16.2,53.7)	27.7 (17.4,43.9)	22.3 (13.6,36.5)
	4 weeks post boost	88.6 (65.3,120.4)	76.5 (53.8,108.8)	120.5 (72.8,199.5)	83.8 (49.3,142.4)	65.8 (46.4,93.3)	70.3 (42.9,115.1)
***B/Brisbane/60/2008*** *Represented in DNA Vaccine Prime*	baseline	25.3 (19.9,32.2)	20.6 (16.0,26.7)	27.1 (18.4,39.8)	29.5 (20.2,43.0)	23.7 (17.3,32.4)	14.1 (10.4,19.3)
	pre-boost	18.1 (13.9,23.6)	16.1 (12.3,21.1)	22.4 (15.4,32.4)	22.3 (15.1,32.8)	14.8 (10.1,21.6)	11.6 (8.1,16.6)
	4 weeks post boost	26.6 (20.7,34.2)	23.4 (18.3,30.0)	30.6 (21.1,44.3)	25.8 (17.3,38.4)	23.3 (16.4,33.1)	21.3 (15.5,29.4)
***A/Victoria/361/2011 (H3N2)*** *Represented in IIV3 Boost*	baseline	125.6 (91.9,171.5)	92.6 (66.6,128.8)	143.5 (88.8,231.7)	104.2 (61.6,176.2)	110.4 (72.3,168.4)	81.8 (54.1,123.6)
	pre-boost	78.0 (55.2,110.2)	64.4 (45.7,90.7)	94.2 (55.8,159.1)	71.7 (40.4,127.3)	64.9 (40.4,104.3)	57.8 (38.5,86.8)
	4 weeks post boost	203.7 (154.5,268.6)	184.9 (131.9,259.2)	237.4 (152.4,369.7)	193.8 (112.8,332.9)	175.7 (123.6,249.8)	176.9 (113.6,275.6)
***B/Wisconsin/1/2010*** *Represented in IIV3 Boost*	baseline	10.4 (8.3,13.1)	10.0 (7.8,12.8)	11.9 (8.4,16.8)	13.3 (8.8,20.2)	9.2 (6.7,12.6)	7.4 (5.9,9.3)
	pre-boost	10.3 (8.3,12.9)	10.2 (7.8,13.4)	10.2 (7.3,14.2)	11.4 (7.6,17.1)	10.4 (7.6,14.3)	9.2 (6.3,13.3)
	4 weeks post boost	25.8 (19.7,33.8)	20.9 (15.6,28.0)	31.3 (21.5,45.5)	25.8 (15.8,42.2)	21.3 (14.3,31.8)	17.2 (12.3,24.0)

The frequency of positive immune response to the prime-boost regimen for the five strains included in the DNA prime (2011/12 seasonal strains) and/or TIV boost (2012/13 seasonal strains) is summarized in Table 5. Response rates following the IIV3 boost were similar between the DNA-prime and PBS-control groups. Within each age stratum (18–50, 51–70) the response rates between treatment groups were similar, but for the three influenza A strains the magnitude was higher in the younger age stratum (Table 6). Results of a post-hoc linear regression analysis showed a trend of higher HAI response following the boost in the younger age stratum, although the difference was only statistically significant for A/California/07/2009 (H1N1) [HAI 2.3 times higher (95% CI 1.5, 3.4); p = 0.0001], and B/Wisconsin/1/2010 [HAI 1.5 times higher (95% CI 1.0, 3.4); p = 0.046]. A summary of immune response magnitude for subjects stratified by the magnitude of pre-existing immune responses is provided in Table 7. Overall, subjects with a positive response at baseline had higher responses at 4 weeks following the boost, but regardless of baseline response, observed responses were similar between the groups receiving DNA or PBS prime.

**Table 7:** GMT 4 weeks Post-Boost for Study Groups by Baseline Immune Response: GMT (95% CI).

	Baseline titer <10				Baseline titer ≥ 10			
	DNA-IIV3		PBS-IIV3		DNA-IIV3		PBS-IIV3	
Antigen	N	GMT	N	GMT	N	GMT	N	GMT
California/04/2009 (H1N1) Represented in DNA Vaccine Prime and IIV3 Boost	4	40.0 (16.3–98.4)	1	10.0 (—)	59	175.0 (132.1–231.7)	61	166.1 (118.0–233.7)
A/Perth/16/2009 (H3N2) Represented in DNA Vaccine Prime	7	26.9 (8.0–91.1)	11	37.6 (13.4–105.2)	56	102.9 (76.1–139.1)	51	89.2 (61.6–129.2)
B/Brisbane/60/2008Represented in DNA Vaccine Prime	8	7.7 (3.4–17.4)	12	9.4 (5.7–15.8)	55	31.9 (25.2–40.3)	50	29.1 (22.6–37.4)
A/Victoria/361/2011 (H3N2) Represented in IIV3 Boost	0	-	1	40.0 (—)	63	203.7 (154.5–268.6)	61	189.6 (135.0–266.2)
B/Wisconsin/1/2010Represented in IIV3 Boost	36	17.8 (12.7–25.0)	37	14.0 (9.7–20.2)	27	42.1 (28.6–62.0)	25	37.8 (25.7–55.7)
	**Baseline titer <40**				**Baseline titer ≥ 40**			
	**DNA-IIV3**		**PBS-IIV3**		**DNA-IIV3**		**PBS-IIV3**	
**Antigen**	**N**	**GMT**	**N**	**GMT**	**N**	**GMT**	**N**	**GMT**
California/04/2009 (H1N1) Represented in DNA Vaccine Prime and IIV3 Boost	11	42.6 (26.2–69.3)	17	47.7 (23.3–97.8)	52	210.6 (160.8–275.9)	45	250.0 (181.8–343.7)
A/Perth/16/2009 (H3N2) Represented in DNA Vaccine Prime	23	36.5 (24.1–55.5)	28	37.1 (21.2–64.9)	40	147.5 (105.7–206.0)	34	138.7 (97.2–198.1)
B/Brisbane/60/2008Represented in DNA Vaccine Prime	38	17.6 (13.1–23.7)	44	16.3 (12.5–21.3)	25	49.9 (36.4–68.6)	18	56.6 (43.1–74.2)
A/Victoria/361/2011 (H3N2) Represented in IIV3 Boost	9	50.4 (29.6–85.9)	14	85.4 (44.3–164.6)	54	257.2 (197.1–335.5)	48	231.6 (158.4–338.5)
B/Wisconsin/1/2010Represented in IIV3 Boost	51	21.1 (15.7–28.4)	52	18.5 (13.4–25.5)	12	59.9 (38.6–92.9)	10	40.0 (21.6–74.2)

## Discussion

We demonstrated in our previous clinical studies targeting H5N1 influenza that a DNA vaccine prime administered 12–24 weeks prior to MIV boost increased the magnitude of protective immune response by 4–6 fold [7, 8, 10]. The population studied in the H5 DNA prime-MIV boost trials was naïve to H5N1 influenza. In the VRC 701 study we tested a DNA prime-IIV3 boost regimen where the DNA vaccine encoded the HAs of all three 2011/12 seasonal influenza strains and 36 weeks later a IIV3 vaccine boost representing the 2012/13 season strains was administered. This regimen was compared to PBS prime-IIV3 boost on the same schedule. The H3N2 and influenza B vaccine strains were new for the 2012/13 season, and the only common influenza strain between the 2011/12 DNA vaccine prime and the 2012/13 IIV3 boost was a pandemic A/California/07/2009(H1N1) which had been circulating for 2–3 years at the time the study began. In addition, to provide a common immunologic baseline, all subjects received the licensed 2011/12 IIV3 at least 8 weeks before enrollment.

The DNA prime—IIV3 boost regimen was safe and well tolerated. Local reactogenicity was predominantly mild. The investigational DNA vaccine was associated with an increased frequency of local pain/tenderness compared to placebo or IIV3, but no differences were observed in other local or systemic reactogenicity symptoms.

Upon analysis of immune responses, we did not detect any significant difference in response rate or magnitude to the 5 influenza strains between the DNA-IIV3 and PBS-IIV3 treatment groups at 4 weeks post boost. The findings in this study do not replicate those of the H5 influenza prime-boost studies. Whereas H5 is an antigen to which there is no prior immunity in study population, the circumstances with seasonal influenza antigens are different. In this case the inability to recognize an impact of DNA priming may be due to 1) immunological priming by previous natural influenza infections or prior vaccinations including the seasonal 2011/2012 IIV3 given just prior to enrollment may have established a repertoire that did not recognize the HA antigens produced by the DNA (original antigenic sin); 2) the mismatched strains in the 2011/12 DNA and the 2012/13 IIV3, or 3) the lengthy 36 week interval covering two influenza seasons. There was a high level of baseline immunity in the adult population with about 70% of the study subjects reporting receipt of seasonal IIV3 3–5 times in the prior 5 years (Table 3).

An interesting but not unexpected finding in this study was that the magnitude of responses in both groups was higher in the younger group of participants (18–50 years of age vs. 51–70 years of age). This is likely due to age related effects on immune responses [11], and may be compounded by the differential pre-existing immunity in the younger subjects who reported less exposure to the traditional IIV3 in previous years; 79% of subjects 18–51 years of age had received IIV3 at least 3 times in the past 5 years compared with 98% of subjects 51–70 years of age (Table 3, p = 0.0005).

We demonstrated in our previous studies that H5 DNA vaccine can prime for a significantly improved immune response to H5N1 MIV when the boost interval is 12–24 weeks [7, 8, 10]. In further assessments of the generalizability of this finding to seasonal influenza antigens, factors such as pre-existing immunity, prime-boost antigen matching, and an optimal boost interval should be carefully considered. A seasonal influenza DNA prime-IIV3 boost regimen with matched antigens, on a 12–24 week schedule is being evaluated. There remains the possibility that the best application of DNA prime-IIV3 boost strategy would be in pre-pandemic situations with more novel influenza strains to which there is low or no pre-existing immunity in the population. This priming strategy may also be applicable to young children for whom several vaccinations with IIV3 are currently needed to induce protective immunity.

## Supporting Information

S1 Protocol VRC 701 Clinical Trial Protocol. (PDF)

S1 CONSORT Checklist CONSORT Checklist. (DOC)
