# Supplementary material for: DNA Priming for Seasonal Influenza Vaccine: A Phase 1b Double-Blind Randomized Clinical Trial
Source: PLoS One. 2015 May 7;10(5):e0125914. doi: 10.1371/journal.pone.0125914 (PMC4423975; doi:10.1371/journal.pone.0125914)
Supplement: S1 Protocol — (PDF) [file pone.0125914.s001.pdf]

A Double-Blind, Randomized Phase 1b Study of the Safety and Immunogenicity of a Prime-Boost Schedule of the 2011/12 Investigational DNA Trivalent Influenza Vaccine, VRC-FLUDNA061-00-VP, Followed by the 2012/13 Seasonal Influenza Trivalent Inactivated Vaccine (TIV) Compared to 2012/13 TIV Alone in Healthy Adults Ages 18-50 and 51-70 Years

Protocol VRC 701

Sponsored by:
National Institute of Allergy and Infectious Diseases (NIAID)
Vaccine Research Center (VRC)
Bethesda, Maryland

BB-IND 13939 – held by VRC, NIAID, NIH

NIAID Funding Mechanism:
Contract # HHSN272201000049I

Clinical Trial Management:
Universal Influenza Clinical Program Support Center (UNI-CPSC)
The EMMES Corporation
Rockville, MD 20850

Version 2.0
March 8, 2012

Confidentiality Statement

This document is confidential and is to be distributed for review only to investigators, potential investigators, consultants, study staff, and applicable independent ethics committees or institutional review boards. The contents of this document shall not be disclosed to others without written authorization from NIAID (or others, as applicable), unless it is necessary to obtain informed consent from potential study participants.

Statement of Compliance

The trial will be conducted in compliance with the protocol, the applicable regulatory requirements including but not limited to the U.S. Code of Federal Regulations applicable to clinical studies (45 CFR 46, 21 CFR including parts 50 and 56 concerning informed consent and IRB regulations, and 21 CFR 312 concerning Investigational New Drug (IND) application), International Conference on Harmonization Good Clinical Practice (ICH-GCP) guidance, and the NIAID Clinical Terms of Contract Award. The site will hold a current Federal Wide Assurance (FWA) issued by OHRP for federally funded research. Completion of Protection of Human Subjects Training will be required for all study personnel in accordance with NIH policy.

TABLE OF CONTENTS

INVESTIGATOR OF RECORD PROTOCOL SIGNATURE PAGE........6
ABBREVIATIONS........7
PRÉCIS........8
1 Introduction........10
1.1 Influenza Biology, Natural History and Vaccines........10
1.2 Rationale for Seasonal Influenza DNA Vaccine Prime-Inactivated Boost........11
1.3 Previous Human Experience with VRC DNA Vaccines........12
1.3.1 Plasmid DNA Vaccines Developed by VRC, NIAID, NIH........12
1.3.2 Influenza DNA Vaccines Developed by VRC, NIAID, NIH........12
1.4 Assessment of Immunogenicity........15
2 Study Vaccines........16
2.1 Formulation and Manufacturing of VRC-FLUDNA061-00-VP........16
2.2 Formulation and Manufacturing of Seasonal Trivalent Influenza Vaccine........17
2.3 Pre-Clinical Studies of HA DNA Vaccines........17
2.3.1 Preclinical Immunogenicity of Influenza Prime-Boost Regimens........17
3 Study Objectives........19
3.1 Primary objective:........19
3.2 Secondary objective:........19
3.3 Exploratory objectives:........19
4 Study Design........19
4.1 Study Population........20
4.1.1 Inclusion Criteria........20
4.1.2 Exclusion Criteria........21
4.2 Study Schedule........22
4.2.1 Pre-enrollment........23
4.2.2 Screening Visit(s)........23
4.2.3 Vaccination Enrollment Visit........23
4.2.4 Administration of Injections:........23
4.2.5 Study Visits and Sample Collection........24
4.3 Concomitant Medications and Procedures........24
4.4 Criteria for Discontinuing Subject Participation........24
4.5 Criteria for Pausing the Study........25
5 Safety and Adverse Events........25
5.1 Adverse Events........25
5.1.1 Adverse Event (AE) Definition........25
5.1.2 Adverse Event Reporting in the Study Database........25
5.2 Serious Adverse Events........26
5.2.1 Serious Adverse Event Definition........26
5.2.2 Reporting Serious Adverse Events to the IND Sponsor........26
5.2.3 IND Sponsor Reporting to the FDA........27

5.3 Reporting to Site IRBs 27
5.4 Data and Safety Monitoring Board 27
6 Statistical Considerations and Sample Analysis 28
6.1 Overview 28
6.2 Objectives 28
6.3 Endpoints 28
6.3.1 Primary Endpoints: Safety 28
6.3.2 Secondary Endpoints: Immune Responses 28
6.3.3 Exploratory Endpoints: Immune Responses 29
6.4 Sample Size and Accrual 29
6.4.1 Power Calculations for Evaluation of Safety 29
6.4.2 Power Calculations for Evaluation of Immune Responses 31
6.4.3 Power Calculations for Immunogenicity Comparisons 31
6.5 Statistical Analysis 32
6.5.1 Analysis Variables 32
6.5.2 Baseline Demographics 32
6.5.3 Safety Analysis 32
6.5.4 Analysis of Immune Responses 33
6.5.5 Interim Analyses 33
6.5.6 Randomization of Treatment Assignments and Unblinding Criteria 34
7 Pharmacy and Vaccine Administration Procedures 35
7.1 Study Agents 35
7.2 Study Agent Presentation and Storage 35
7.2.1 Study Agent Labels 35
7.2.2 Study Agent Storage 35
7.3 Preparation of Study Agent for Injection 36
7.3.1 Preparation of VRC-FLUDNA061-00-VP 36
7.3.2 Preparation of Phosphate Buffered Saline (PBS) 36
7.3.3 Preparation of 2012-2013 TIV Injection 37
7.4 Study Agent Accountability 37
7.4.1 Documentation 37
7.4.2 Disposition 37
8 Human Subjects Protection 37
8.1 Institutional Review Board 37
8.2 Subject Recruitment and Enrollment 37
8.2.1 Participation of Children 37
8.3 Informed Consent 38
8.4 Subject Confidentiality 38
8.5 Risks and Benefits 38
8.5.1 Risks of Blood Collections: 38
8.5.2 Risks of the DNA Vaccine: 38
8.5.3 Risks of the Seasonal TIV Vaccine: 39
8.5.4 Other Risks: 40
8.5.5 Benefits: 40
8.6 Plan for Use and Storage of Biological Samples 40

8.7 Compensation 41
8.8 Safety Monitoring 41
8.8.1 Protocol Safety Review Team 41
8.8.2 DSMB 41
9 Administration and Legal Obligations 41
9.1 Protocol Initiation, Amendments and Termination 41
9.2 Study Documentation and Study Records Retention 41
9.3 Data Collection and Protocol Monitoring 42
9.3.1 Data Capture Methods 42
9.3.2 Source Documents and Access to Source Data/Documents 42
9.3.3 Protocol Monitoring 42
9.4 Language 43
9.5 Policy Regarding Research-Related Injuries 43
10 References 44
Appendix I: Template Informed Consent Forms 47
Appendix II: Contact Information 55
Appendix III: Schedule of Evaluations 57
Appendix IV: Assessment of Relationship to Vaccine and Adverse Event Severity Grading 59

INVESTIGATOR OF RECORD PROTOCOL SIGNATURE PAGE
VRC 701

A Double-Blind, Randomized Phase 1b Study of the Safety and Immunogenicity of a Prime-Boost Schedule of the 2011/12 Investigational DNA Trivalent Influenza Vaccine, VRC-FLUDNA061-00-VP, Followed by the 2012/13 Seasonal Influenza Trivalent Inactivated Vaccine (TIV) Compared to 2012/13 TIV Alone in Healthy Adults Ages 18-50 and 51-70 Years

Sponsored by:
Vaccine Research Center, National Institute of Allergy and Infectious Diseases
National Institutes of Health

I, the Investigator of Record for the indicated Study Site, agree to conduct this study in full accordance with the provisions of this protocol. I agree to maintain all study documentation pertaining to the conduct of this study, including CRFs, source documents, consent forms, laboratory test results, and medication inventory records, for at least 2 years following submission of a Biologics License Application, unless directed otherwise by the VRC. No study records will be destroyed without prior authorization from NIAID. Publication of the results of this study will be governed by the VRC and NIAID policies. Any presentation, abstract, or manuscript will be made available by the investigators to the VRC Leadership Group and to NIAID for review prior to submission.

I have read and understand the information in this protocol and will ensure that all associates, colleagues, and employees assisting in the conduct of the study are informed about the obligations incurred by their contribution to the study.

______________________________ ______________________________
Name /Title of Investigator of Record Study Site Name/Identifier

______________________________ ______________________________
Signature of Investigator of Record Date

ABBREVIATIONS

Abbreviation	Term
AE	adverse event
CBC	complete blood count
CC	Clinical Center
CDC	Center for Disease Control
CRO	Contract Research Organization
DNA	deoxyribonucleic acid
DSMB	Data and Safety Monitoring Board
EDC	Electronic data capture
ELISA	enzyme-linked immunosorbent assay
ELISpot	enzyme-linked immunospot
FDA	Food and Drug Administration
GCP	Good Clinical Practices
HA	Hemagglutinin
HAI	Hemagglutination Inhibition
HIV	human immunodeficiency virus
HLA	human leukocyte antigen
ICS	intracellular cytokine staining
IDES	Internet Data Entry System
IoR	Investigator of Record
IRB	Institutional Review Board
ITT	Intent-to-treat
LIMS	Laboratory Information Management System
MedDRA®	Medical Dictionary for Regulatory Activities
NA	Neuraminidase
NH	Northern Hemisphere
NIAID	National Institute of Allergy and Infectious Diseases
NIH	National Institutes of Health
NVITAL	NIAID Vaccine Immune T-Cell and Antibody Laboratory
PBMC	peripheral blood mononuclear cells
PBS	Phosphate-buffered saline
RNA	ribonucleic acid
SAE	serious adverse event
SH	Southern Hemisphere
S-OIV	Swine-Origin Influenza Virus
TIV	trivalent inactivated vaccine (for seasonal influenza)
UNI-CPSC	Universal Influenza Clinical Program Support Center
VAERS	Vaccine Adverse Events Reporting System
VIS	vaccine information statements
VRC	Vaccine Research Center
WHO	World Health Organization

PRÉCIS

Protocol VRC 701: A Double-Blind, Randomized Phase 1b Study of the Safety and Immunogenicity of a Prime-Boost Schedule of the 2011/12 Investigational DNA Trivalent Influenza Vaccine, VRC-FLUDNA061-00-VP, Followed by the 2012/13 Seasonal Influenza Trivalent Inactivated Vaccine (TIV) Compared to 2012/13 TIV Alone in Healthy Adults Ages 18-50 and 51-70 Years

Study Design: This is a Phase 1b, randomized study in healthy younger (18-50 years) and older (51-70 years) adults to evaluate the safety, tolerability, and immunogenicity of a prime-boost vaccination regimen with an investigational plasmid DNA vaccine directed towards the 2011/12 influenza vaccine strains as a prime followed 36 weeks later by the 2012/13 influenza trivalent inactivated vaccine (TIV) boost, as compared to placebo prime followed by the 2012/13 seasonal TIV. The hypothesis is that the DNA vaccine will be safe for human administration and that the prime-boost schedule will elicit a higher titer antibody response than the seasonal TIV alone.

Study Objectives: The primary objectives are to evaluate the safety and tolerability and induced antibody titer of the investigational prime-boost regimen, at a dose of 4 milligrams (mg) for the DNA vaccine and 45 micrograms (mcg) for the seasonal TIV vaccine. Secondary and exploratory objectives are related to the humoral and cellular immune responses.

Product Description: The VRC-FLUDNA061-00-VP vaccine was developed and manufactured by VRC, NIAID as an investigational 2011-2012 influenza DNA vaccine and is composed of 3 closed-circular DNA plasmids, each with a CMV/R promoter that encode for the hemagglutinin (HA) from the following 3 strains: A/California/04/2009 (H1N1); A/Perth/16/2009 (H3N2), and B/Brisbane/60/2008. DNA vaccine vials will be supplied at 4 mg/mL and each dose will be 1 mL. The placebo for the DNA vaccine is phosphate buffered saline (PBS). DNA and PBS injection will be administered intramuscularly (IM) in deltoid muscle using the Biojector® 2000 Needle-Free Injection Management System (Biojector). The inactivated seasonal influenza vaccine will be a standard licensed trivalent subunit vaccine for the 2012/13 season. Each dose will be composed of 45 mcg HA in 0.5 mL with the recommended ratio of 15 mcg HA of each of three strains [A/California/7/2009 (H1N1)pdm09-like, A/Victoria/361/2011 (H3N2)-like and B/Wisconsin/1/2010-like] selected.

Subjects: The target accrual is 120 healthy adults; 60 in the 18-50 year age group and 60 in the 51-70 year age group. Enrollment of up to 66 per age group is permitted. To be eligible to participate, subjects must have received a 2011/12 FDA-licensed influenza vaccine at least 8 weeks before the Day 0 study injection.

Study Plan: The randomization in the study is stratified by age. Subjects in each age group will be randomized at a ratio of 1:1 to either the DNA prime-TIV boost schedule or the placebo prime-TIV schedule.

The Day 0 injections will be given during the 2011/12 influenza season and the 2012/13 TIV injections about 36 weeks later during the 2012/13 influenza season.

**Protocol VRC 701**			**Injection Schedule**	
**Group**	**Subjects Enrolled**	**Blinded Randomization**	**Day 0**	**Week 36 (±4 weeks)**
Group 1<br>18-50 years	60	Group 1A: n=30	DNA IM Biojector	TIV IM Needle
		Group 1B: n=30	PBS IM Biojector	TIV IM Needle
Group 2<br>51-70 years	60	Group 2A: n=30	DNA IM Biojector	TIV IM Needle
		Group 2B: n=30	PBS IM Biojector	TIV IM Needle
TOTAL	120 subjects		Day 0 injection is blinded;<br>TIV vaccination is open-label	

Study Duration: The protocol requires 7 clinic visits (Screening, Day 0, Week 4, Week 24, Week 36, Week 40, and Week 60) and 2 telephone follow-up contacts for all groups. Each subject will complete follow up through 24 weeks after the TIV injection. The expected duration of time on study per subject is approximately 60 weeks.

1 INTRODUCTION

The Dale and Betty Bumpers Vaccine Research Center (VRC), NIAID, NIH (Bethesda, MD) is dedicated to translating the latest knowledge of disease pathogenesis and immunology into new vaccine strategies to provide safe and effective means to prevent and control infectious diseases. The 2009-2010 H1N1 pandemic influenza reinforces awareness that rapidly preparing a vaccine for use during an influenza season and consideration of the different levels of immunity and risks present in different age groups in the population is important for public health [1]. The need for influenza vaccines that are both more immunogenic and able to induce universal immune responses effective against a broad spectrum of influenza strains is well recognized. In this protocol we propose to use gene-based vaccine antigen delivery to induce immune responses against native hemagglutinin (HA) structures prior to boosting with traditional licensed split virus trivalent influenza vaccine (TIV). There is pre-clinical evidence suggesting this approach will contribute to understanding how to develop influenza vaccines that induce immunity that is cross-reactive against old, new and re-emerging influenza strains and with improved durability of response.

1.1 INFLUENZA BIOLOGY, NATURAL HISTORY AND VACCINES

Influenza is a negative-strand ribonucleic acid (RNA) virus with a segmented genome that belongs to the family *Orthomyxoviridae*. Of the three genera of influenza circulating in nature (influenza A, B, and C), only the first two are known to cause epidemics [2]. Influenza A viruses have 8 open reading frames that encode 10 viral proteins. They are classified on the basis of the antigenicity of their surface glycoproteins: hemagglutinin (HA) and neuraminidase (NA). Sixteen HA subtypes and 9 NA subtypes are known to exist, but only three HA subtypes (H1, H2, and H3) and two NA subtypes (N1 and N2) have caused significant human epidemics [3].

The public health burden of influenza in the world is enormous. Annual influenza epidemics cause about 250,000 to 500,000 deaths worldwide [4]. Circulating viruses change quickly and re-assort with each other creating new viruses. These present an immediate threat to public health and currently require the preparation of new vaccines directed at the changing viral strains that are prevalent annually. Emerging virus strains present the potential of a pandemic when there is little or no pre-existing immunity in the population as observed in worldwide outbreaks of influenza in the last century [5]. A global pandemic caused by spread of a novel swine-origin influenza virus (S-OIV) in 2009-2010 influenza season is a recent example of this challenge to public health [6].

Vaccines are an effective way of preventing influenza infection and transmission in humans. Annually, the World Health Organization (WHO) and the U.S FDA make recommendations on the composition of the seasonal influenza vaccine, with recommendations for the Northern Hemisphere (NH) and for the Southern Hemisphere (SH) considered at different times based on epidemiology data [7]. The annually licensed influenza vaccines consist of 3 components: Influenza A (H1N1), Influenza A (H3N2), and an influenza B virus strain. These vaccines depend upon labor-intensive methods that limit manufacturing capacity and have low immunogenicity to induce type-specific responses. Currently, the vaccine composition requires an adjustment for emerging antigenically-modified influenza strains and efficacy is limited in vulnerable populations.

For seasonal influenza infection, the rates of serious illness and death are the highest among persons older than 65 years, children of less than 2 years old, and persons of any age who have

medical conditions that place them at increased risk for complications from influenza [8]. Seasonal influenza causes significantly higher morbidity and mortality in older adults than in younger adults and these adverse outcomes are attributed to an age-related decline in immune function [9].

Seasonal TIVs have demonstrated a clinical efficacy of 70% to 90% in younger adults, ~ 58% in those aged 60 to 69 years, and 30% to 40% in those aged 70 or more years [10]. When the seasonal vaccine and circulating viruses are antigenically similar, seasonal TIV prevents laboratory-confirmed influenza illness among approximately 70%-90% of healthy adults less than 65 years old in randomized controlled trials. Immunization efficacy was 47%-77% in studies conducted during influenza seasons when the vaccine strains were not an exact antigenic match to the majority of circulating influenza strains [8].

In the 2009-2010 H1N1 influenza pandemic, the CDC determined that the seasonal TIV was unlikely to provide protection against a novel pandemic influenza A (H1N1) that was an antigenically distinct influenza virus to which little or no pre-existing immunity was detected in population [1]. The epidemiologic pattern of this novel swine-origin influenza virus (S-OIV) strain included a high prevalence (60% of infected patients) in the population ≤ 18 years old, who had not been exposed to the influenza strains that circulated in 1950-1970s that had partial antigenic similarity with the novel H1N1 strain [1, 11]. Using stored serum specimens collected in previous vaccine studies, CDC assessed the level of cross-reactive antibodies to the novel influenza A (H1N1) virus in cohorts of children and adults before and after they had been vaccinated with the 2005-2009 influenza seasonal vaccines [1]. The results indicated that 33% of adults older than 60 years and 6-9% of adults aged 18-64 years had some cross-reactive antibody to the novel influenza, and no cross-reactive antibody was found in children. The results also suggested that seasonal influenza vaccines are unlikely to provide protection against novel emerging influenza virus strains. Development of a universal influenza vaccine that will have an improved cross-reactivity and efficacy against novel emerging influenza strains, as well as high potency in the vulnerable high-risk populations is a priority for public health in the United States and worldwide [12].

Several conserved regions that may serve as broad neutralizing epitopes to afford cross-protection against a variety of influenza strains have been identified in the influenza viral protein structures [13-15]. NIAID scientists have been engaged in investigation of immune responses to influenza and identification of the broadly-neutralizing antibodies that may lead to a development of a more universal influenza vaccine [15, 16]. The DNA vaccine prime-inactivated vaccine boost strategy being evaluated in this protocol has shown evidence of improved immune response, including to epitopes conserved between influenza subtypes. The rationale for and prior experience with this strategy is summarized below.

1.2 Rationale for Seasonal Influenza DNA Vaccine Prime-Inactivated Boost

DNA vaccines have the potential to be manufactured rapidly. They are known to induce balanced immune responses that induce both humoral and cellular immunity. The potential and experience to date with influenza DNA vaccines warrants continued investigation. One approach to improving immunity to influenza would be to prime the population with a DNA vaccine early in the year and to boost with TIV when the influenza season begins in order to provide better immunogenicity, especially to novel influenza strains. This may be a useful strategy for the older adult and pediatric populations for which the TIV vaccine alone has a lower efficacy than in

young adults. In previous studies, the VRC has found that antibody responses are higher after the boost when there is a long prime-boost interval, such as 6 months compared to a short interval, such as 1 month. For this reason, one dose DNA priming schedules with a long interval between prime and boost are being evaluated towards the goal of an influenza vaccination strategy with improved immunogenicity.

A DNA vaccine may also elicit CD8 T cell responses to conserved HA epitopes that may afford some cross-protection [17-19]. It has been also suggested that T cell responses are the important correlates of influenza vaccine protection, especially in the elderly populations immunized with the seasonal flu vaccines [20, 21].

1.3 PREVIOUS HUMAN EXPERIENCE WITH VRC DNA VACCINES

1.3.1 Plasmid DNA Vaccines Developed by VRC, NIAID, NIH

Investigators at the VRC/NIAID/NIH in Bethesda, MD have evaluated plasmid DNA vaccine strategies since 2001 in preclinical and clinical studies. Cumulatively, worldwide about 2000 study subjects have been vaccinated with a VRC DNA vaccine. Dosages up to 8 mg have been administered, with the majority of injections being at a 4 mg dosage. Data from dose-escalation studies indicate that a 4 mg dosage offers the combination of a good safety profile, greater ease of administration than an 8 mg dosage, and reliable immunogenicity as indicated by laboratory measures of immune response. Preclinical and clinical evaluations to date of several plasmid DNA vaccines support the safety and immunogenicity of VRC DNA vaccines at the 4 mg dosage. The results of human clinical trials with VRC DNA vaccines have been published for HIV DNA vaccines [22-27], an Ebola virus DNA vaccine [28], a SARS DNA vaccine [29], West Nile virus DNA vaccines [30, 31] and H5 DNA vaccine [32]. VRC clinical trials of WNV and SARS vaccines have provided evidence that a DNA vaccine can induce neutralizing antibody as assessed by a reporter virus particle (RVP) neutralizing antibody assay [29-31]. Experience to date indicates that there may be advantages to using a Biojector for DNA vaccine delivery compared to using a standard needle and syringe [33]. IM and SC delivery of vaccines via Biojector is cleared for use by the FDA in adults and children.

1.3.2 Influenza DNA Vaccines Developed by VRC, NIAID, NIH

The VRC, NIAID, NIH has developed several investigational influenza DNA vaccine products. Clinical evaluation of the first influenza DNA product was initiated in 2006 with an avian influenza H5 DNA vaccine. Through a series of clinical trials, influenza DNA vaccines have been tested alone or in prime-boost regimens with the respective inactivated vaccines in the clinical trials as shown in **Table 1-1**:

Table 1-1: Experience with VRC Influenza DNA Vaccines in Adult Population

IND Number	Vaccine(s)	Antigen(s)	Protocols
BB-IND 13197	VRC-AVIDNA036-00-VP	HA of A/Indonesia/05/2005 (H5N1)	VRC 304<br>VRC 305
BB-IND 13836	VRC-AVIDNA036-00-VP<br>(with inactivated vaccine boost)	HA of A/Indonesia/05/2005 (H5N1)	VRC 306<br>VRC 310
BB-IND 13939	VRC-FLUDNA047-00-VP<br>(with inactivated vaccine boost)	Trivalent HA matching the 2008-2009 seasonal strains	VRC 307
	VRC-FLUDNA056-00-VP<br>(with inactivated vaccine boost)	Trivalent HA matching the 2009-2010 seasonal strains	VRC 309
BB-IND 14093	VRC-FLUDNA057-00-VP<br>(with inactivated vaccine boost)	HA of pandemic A/California/04/2009 (H1N1)	VRC 308

H5 DNA vaccine alone (BB-IND 13197) was evaluated in two Phase 1 studies. One study (VRC 304) investigated intramuscular (IM) administration of the vaccine or placebo in three groups of healthy adults 18-60 years of age who received 3 IM injections of 1 mg vaccine, 4 mg vaccine, or placebo, respectively. All injections in VRC 304 were via Biojector. The second study, VRC 305, investigated intradermal (ID) administration of the vaccine by 4 schedules: 0.5 mg ID by needle, 0.5 mg ID by Biojector, 0.5 mg ID x2 in the same arm, and 0.5 mg ID in each arm. Cumulatively, 89 subjects were enrolled. Between the two studies, 204/222 (92%) of the expected DNA vaccinations were administered to 74 vaccine recipients and 42/45 (93%) of expected phosphate buffered saline (PBS) injections were given to 15 placebo recipients.

The H5 DNA vaccine was well tolerated by both the IM and ID routes. Detailed final study reports are available in the BB-IND 13197. In VRC 304, no apparent dose effect on frequency, duration, or severity of reactogenicity was noted. There were two serious adverse events (SAE) in VRC 304: a Grade 3 leukocytosis, attributed to concomitant medications taken for a musculoskeletal injury and a Grade 3 paroxysmal hemicrania in a subject with a history of severe headaches. A neurologist assessed this as unlikely to be related to vaccine. In VRC 305, there were no SAE.

The H5 DNA vaccine alone was not strongly immunogenic by either the IM or ID routes.

H5 DNA Vaccine in prime-boost regimens (BB-IND 13836) was evaluated in two Phase 1 studies (VRC 306 and VRC 310) in healthy adults ages 18-60 years old. Between these two studies the prime-boost regimens evaluated included two doses of monovalent inactivated vaccine (MIV, Sanofi Pasteur H5N1 A/Indonesia 05/05 avian influenza vaccine) at 4 or 24 week intervals or H5 DNA vaccine followed by MIV at varying intervals from 4 to 24 weeks. Cumulatively, 124 subjects were enrolled. Between the two studies, 114/114 (100%) of the expected H5 DNA injections were administered to 99 subjects randomized to schedules with H5 DNA primes and 147/149 (99%) of the expected H5N1 MIV injections were administered to 122 subjects.

There was no severe local or systemic reactogenicity. Mild local reactogenicity was reported in a

majority of the H5 DNA vaccine recipients (83%) while 2% reported moderate local reactogenicity. The majority of the H5 DNA vaccine recipients reported no systemic reactogenicity; 23% reported mild and 5% reported moderate systemic reactogenicity. No SAE were reported following the H5 DNA vaccinations; there was one SAE reported in the follow-up period through 48 weeks: multiple injuries unrelated to study participation in the period after the H5N1 booster. All adverse events following H5 DNA injection were mild (Grade 1) or moderate (Grade 2) except for one severe (Grade 3) case of gastroenteritis with onset 15 days after H5 DNA vaccination, which was assessed as unrelated to vaccine.

In assessing immunogenicity of H5 DNA vaccine followed by MIV, a single H5 DNA vaccine prime followed by a single MIV boost at short intervals (4-8 weeks) did not significantly improve HAI titers over that achieved with inactivated vaccine alone. However, a single H5 DNA 4 mg vaccination prime significantly improved HAI responses when the interval to the inactivated vaccine boost was 20-24 weeks, as compared with two vaccinations with the inactivated vaccine [32]. Therefore, a single dose of DNA priming may be sufficient to increase the magnitude and breadth of HA-specific antibody responses.

<u>Seasonal HA DNA vaccine studies (BB-IND 13939 and BB-IND 14093)</u>: Specifically, with regard to seasonal influenza vaccines developed by the VRC/NIAID/NIH, VRC-FLUDNA047-00-VP, VRC-FLUDNA056-00-VP, and VRC-FLUDNA061-00-VP are season-specific 3-plasmid DNA vaccines; while VRC-FLUDNA057-00-VP is a single plasmid H1 DNA based on the 2009 pandemic H1N1 influenza virus. **Table 1-2** shows the composition of these investigational influenza DNA vaccines.

Table 1-2: Composition of VRC Seasonal Influenza DNA Vaccine Products

	Plasmids and Expressed Antigens					
	VRC-9269	VRC-9328	VRC-9270	VRC-2439	VRC-9271	VRC-9323
	H1	H1	H3	H3	B	B
Product	A/Brisbane /59/07	A/California /04/09	A/Brisbane /10/07	A/Perth/ 16/09	B/Florida/ 4/06	B/Brisbane / 60/08
VRC-FLUDNA047-00-VP Seasonal 2008-09	X		X		X	
VRC-FLUDNA056-00-VP Seasonal 2009-10	X		X			X
VRC-FLUDNA057-00-VP Pandemic H1 2009		X				
VRC-FLUDNA061-00-VP Seasonal 2011-12		X		X		X

Three clinical trials have been completed in adults with seasonal HA DNA vaccines. VRC 307 (2008-2009 seasonal) and VRC 309 (2009-2010 seasonal) were Phase 1 clinical trials to evaluate trivalent seasonal HA DNA constructs in adults 18-70 years old. VRC 308 was a Phase 1 clinical trial to evaluate a 1-plasmid construct for the 2009 pandemic H1 influenza in adults 18-70 years old. The prime-boost interval of VRC 307 and VRC 309 was 3 or 4 weeks, while for VRC 308, due to delayed availability of the pandemic H1N1 inactivated vaccine, the boost was added by amendment to be offered to subjects as an option, and the boost intervals varied.

In VRC 307 and VRC 309, the DNA constructs were the same except for the encoded influenza B antigen. Cumulatively, a total of 111 subjects were enrolled; 66 were randomized to a single

HA DNA prime-TIV boost, 25 to a single PBS prime-TIV boost, and 20 to TIV prime-TIV boost schedules. Between the two studies 65/66 (99%) of the expected HA DNA vaccinations, 25/25 (100%) of the expected PBS injections and 126/131 (96%) of the expected TIV vaccinations were administered. The seasonal HA DNA vaccines were well tolerated. There was no severe local or systemic reactogenicity. Similar to the H5 DNA vaccine, a majority of seasonal HA DNA vaccine recipients (78%) experienced mild local reactogenicity and 2% experienced moderate local reactogenicity. The majority (66%) of HA DNA vaccine recipients reported no systemic reactogenicity, while 31% reported, mild and 3% reported moderate systemic reactogenicity. No SAE were reported in either VRC 307 or VRC 309. All adverse events were mild or moderate except for three Grade 3 events following HA DNA prime (urticaria 1 day after vaccination, influenza 11 days after vaccination, and increased alanine aminotransferase [ALT] 33 days after vaccination); one Grade 3 event following PBS prime injection (neutropenia); and one Grade 3 event (gastroenteritis) following TIV. Based on temporal relationship, urticaria was the only Grade 3 event assessed as possibly related to DNA vaccine.

VRC 308 included 20 subjects on a schedule of three H1 DNA vaccinations at 4 week intervals; 60/60 (100%) of expected H1 DNA vaccines were administered and the vaccine was well tolerated. When the licensed H1N1 inactivated vaccine became available, 18 subjects opted to receive the boost as well. There was no severe local or systemic reactogenicity. The majority (90%) of H1 DNA vaccine recipients reported mild local reactogenicity; one subject (5%) reported moderate local reactogenicity. Over 3 injections with H1 DNA vaccine, 60% reported mild and 15% reported moderate systemic reactogenicity. There were no SAE and all adverse events were mild or moderate in severity.

Summary of HA DNA safety and immunogenicity: The safety data for the VRC HA DNA vaccine trials to date shows that these vaccines were well tolerated. No severe local or systemic reactogenicity occurred nor were there any serious adverse events related to vaccine. The pattern of local and systemic reactogenicity appears to be generally mild and similar across the studies, independent of the antigens encoded by the HA DNA constructs included in any one vaccine.

Regarding immunogenicity, seasonal HA DNA priming did not significantly improve HAI response compared to TIV alone with the boost administered at a 3-4 week interval. However, data from studies of the H5 DNA vaccine using various H5N1 inactivated vaccine (MIV) boost intervals show that a longer interval is associated with a higher magnitude of immune response.

Based on experience with the H5 DNA vaccine, future development of the trivalent seasonal HA DNA is directed towards evaluation of an inactivated boost at intervals 20 weeks or longer post-prime as a potential method of reliably inducing a strong immune response to seasonal influenza vaccine.

1.4 ASSESSMENT OF IMMUNOGENICITY

In protocol VRC 701, specimens to evaluate immunogenicity will be taken at baseline and at specified time points. The primary immunogenicity timepoint is 4 weeks after the boost. Measurements of antibody, B cell and T cell response will be assessed. Hemagglutinin (HA)-specific antibody as measured by HAI assay is the traditional benchmark measure of immune response to influenza vaccines and will be conducted on stored samples from throughout the study. To evaluate durability of HA-specific immune responses, the response will be assessed at

4 and 24 weeks after the boost. Because these adult study subjects are likely to have pre-existing immune responses to many influenza hemagglutinin antigens and the effect of vaccination on other humoral and T cell responses is of interest, a variety of exploratory evaluations of immunogenicity may also be performed. HA-specific T cell responses measured by intracellular cytokine staining (ICS) assay and ELISpot, other H5-antibody assays, and assays to evaluate cross-reactivity will be performed at timepoints throughout the study as exploratory evaluations.

The detection of antibody by HAI assay is based on a validated laboratory method. The ICS assay is based upon previously published methods [34] and quantitates the frequency of $CD4^+$ and $CD8^+$ cells that produce interleukin-2, interferon-gamma, or TNF-alpha in response to pools of overlapping peptides representing HA antigens. Specific peptides will also be used to detect T cell responsiveness by an ELISpot assay, modified from a previously published method [35]. Research samples for immunogenicity assays will be processed by the NIAID Vaccine Immune T Cell and Antibody Laboratory (NVITAL) in Gaithersburg, MD, where many of the immunogenicity assays will also be performed. Some immunogenicity assays may be performed by VRC laboratories in Bethesda, MD, approved contract laboratories, or approved research collaborators

2 STUDY VACCINES

2.1 FORMULATION AND MANUFACTURING OF VRC-FLUDNA061-00-VP

The VRC-FLUDNA061-00-VP Drug Substance consists of three closed-circular plasmid DNA macromolecules (VRC-9328, VRC-2439 and VRC-9323), in equal amounts by weight, that express Influenza A HA sequences for strains that meet the criteria for production of the 2011-2012 licensed seasonal influenza vaccine, as follows:

- VRC-9328--HA (Accession #GQ117044)-Influenza A virus (A/California/04/2009) (H1N1),
- VRC-2439--HA (Accession #ACS71642)-Influenza A virus (A/Perth/16/2009) (H3N2),
- VRC-9323--HA (Accession #ACN29380)-Influenza B virus (B/Brisbane/60/2008)

The plasmid CMV/R promoter consists of translational enhancer region of the CMV immediate early region 1 enhancer (CMV-IE) substituted with the 5′-untranslated human T-cell leukemia virus type 1 (HTLV-1) R-U5 region of the human T-cell leukemia virus type 1 HTLV-1 long terminal repeat (LTR), and has been shown to increase expression of the encoded gene in comparison to the CMV promoter [36]. This promoter has been evaluated in preclinical safety studies as well as in many clinical trials.

VRC-FLUDNA061-00-VP is manufactured at the VRC/NIAID/Vaccine Pilot Plant (VPP) using plasmid DNA received from the VRC to produce clinical trial material under current Good Manufacturing Practices (cGMP). The process for manufacturing, filling, and packaging the vaccine is summarized in the Investigator’s Brochure (IB). Briefly, the plasmids used in the Master Cell Banks (MCB) were synthesized using human preferred codons as previously described [37]. The plasmids were then transferred to the VPP and their sequences confirmed before use. Each plasmid was used to transform the *Escherichia coli* bacterial host strain, DH5α, in order to produce individual MCB. Each MCB was expanded in culture and inoculated into a fermentor for production. Bacterial cell growth was dependent upon the cellular expression of

the kanamycin resistance protein encoded by a portion of the plasmid DNA. Following growth of bacterial cells harboring the plasmid, the plasmid DNA was purified from cellular components, concentrated, filtered, and stored until formulation of the drug product. The final vaccine product will meet lot release specifications prior to administration.

The Drug Product is manufactured at a 4 mg dose in phosphate buffered saline (PBS). Vials are aseptically filled to a volume of 1.2 mL with 4 mg/mL plasmid.

2.2 Formulation and Manufacturing of Seasonal Trivalent Influenza Vaccine

Study sites will refer to the manufacturing package insert for complete product information. The TIV used for the booster injection will be a licensed Northern Hemisphere (NH) product approved by the U.S. FDA for administration during the 2012/13 influenza season, composed of three strains: A/California/7/2009 (H1N1)pdm09-like, A/Victoria/361/2011 (H3N2)-like and B/Wisconsin/1/2010-like).

2.3 Pre-Clinical Studies of HA DNA Vaccines

No preclinical pharmacology, toxicology, pharmacokinetic, or metabolism studies were conducted for the seasonal influenza DNA vaccine VRC-FLUDNA061-00-VP. There is extensive clinical experience with licensed seasonal influenza products, including the antigens present in the 2011-2012 seasonal influenza vaccine, as well as human clinical trial experience with DNA vaccines constructed using the same plasmid backbone and CMV/R promoter, including other seasonal influenza DNA vaccines.

FDA recommendations permit manufacturers with established seasonal flu vaccines to file supplemental applications each year after changing one or more of the three influenza strains in the vaccine. This reflects the large safety experience with similar influenza vaccine products as well as the severe time constraints on design and manufacture of both investigational and licensed vaccines due to the seasonality of influenza infections. Although VRC is evaluating investigational vaccines based on the DNA platform, the influenza gene inserts mimic the antigens found in the yearly seasonal TIV and our approach has been similar in this regard.

2.3.1 Preclinical Immunogenicity of Influenza Prime-Boost Regimens

The VRC has conducted non-clinical, non-GLP immunogenicity studies of plasmid DNA influenza vaccines, similar in construction to the trivalent plasmid product VRC-FLUDNA061-00-VP, demonstrating that that vaccination with plasmid DNA encoding HA boosted with seasonal vaccine induces broadly neutralizing influenza antibodies. This was associated with increased neutralization of diverse strains compared to either component alone and protection was demonstrated in mice and ferrets. It was also shown in nonhuman primates that antibodies directed to the conserved stem region of HA were also elicited [16]. Cross-neutralization elicited by this approach provides a basis for the development of a universal influenza vaccine for humans.

Figure 2-1 shows results from the Wei, *et al* published study [16]. In the mouse study, the DNA plasmids used were based on the 2006–2007 vaccine strains: A/New Caledonia/20/99 (1999 NC) (H1N1) and A/Wisconsin/67/05 (2005 WI) (H3N2). Mice were immunized with an empty

plasmid (control) or an HA-encoding plasmid, followed by a boost with 2006–2007 seasonal TIV that expressed matching H1 or H3 HA. Vaccination with 1999 NC H1 DNA followed by 2006–2007 seasonal inactivated vaccine boost stimulated a greater than 50-fold increase in neutralizing antibody titer to H1 HA than that produced by one dose of either the DNA or inactivated vaccine alone. This is shown in Figure 2-1A. To evaluate the breadth of neutralization, antisera were analyzed for their ability to neutralize heterologous H1N1 strains. The DNA/seasonal vaccine antiserum neutralized previous H1N1 strains dating back to 1934, as well as the activity of A/Brisbane/59/2007 (H1N1). This is shown in Figure 2-1B. Priming with 2005 WI H3 DNA vaccine followed by a 2006–2007 seasonal inactivated vaccine boost failed to stimulate an increase in neutralization titer to H1N1 (1999 NC). This is shown in Figure 2-1C.

Fig. 2-1 Increased titer and breadth of neutralizing antibodies to H1N1 strains elicited by DNA/seasonal flu vaccine immunization (C Wei et al. Science 2010;329:1060-1064)

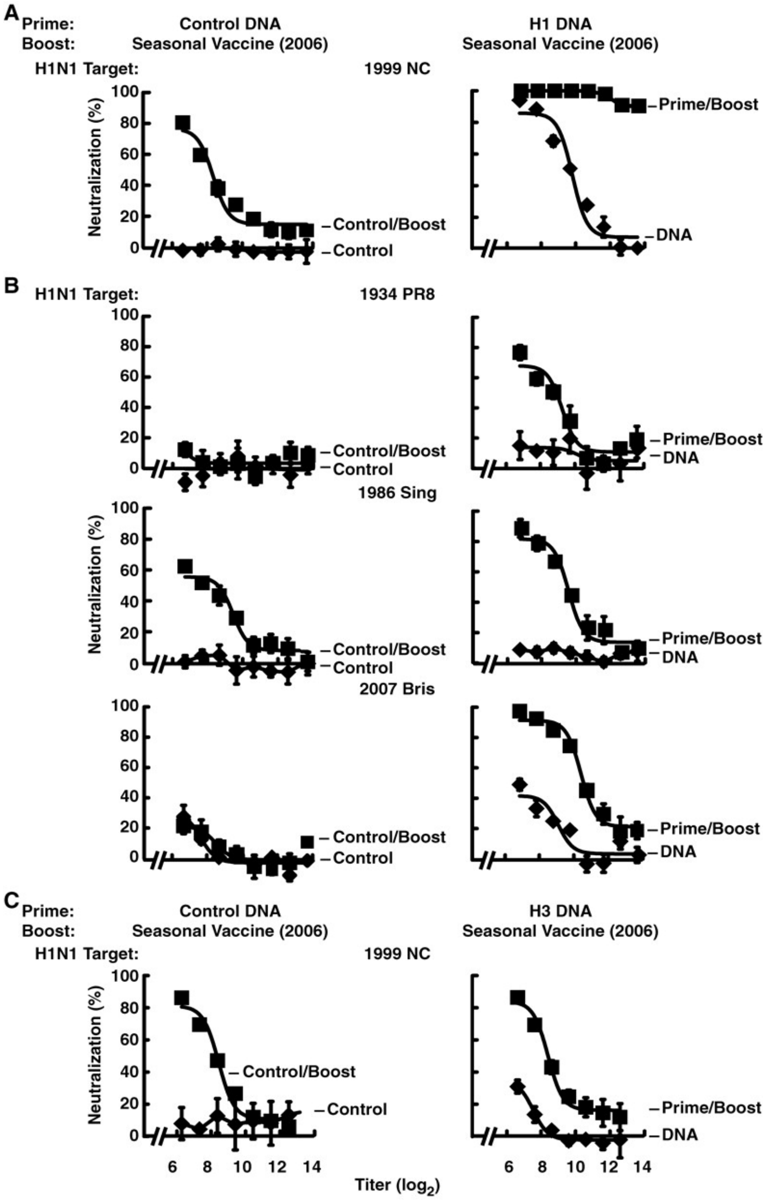

Figure 2-1 Legend: *"Increased titer and breadth of neutralizing antibodies to H1N1 strains elicited by DNA/seasonal flu vaccine immunization. (A) Pseudotyped neutralization assay to measure the neutralizing antibody response in mice immunized with homologous H1N1 1999 NC HA DNA vaccine, seasonal flu vaccine, or a DNA prime and seasonal flu vaccine boost regimen. (B) The neutralizing antibody response in mice against 1934 PR8, A/Singapore/6/1986 (1986 Sing), and 2007 Bris HA-pseudotyped lentivirus reporters after a DNA prime/vaccine boost regimen. (C) The neutralizing antibody response against a 1999 NC HA pseudotyped lentivirus reporter in response to an H3N2 HA DNA (A/Wisconsin/67/2005) prime/seasonal vaccine boost*."

3 STUDY OBJECTIVES

3.1 PRIMARY OBJECTIVE:

- To evaluate in adults age 18-70 years the safety and tolerability of a prime-boost study regimen of the recombinant DNA vaccine VRC-FLUDNA061-00-VP (administered IM with a Biojector at a dosage of 4 mg) followed at week 36 by 2012/13 seasonal influenza TIV, (administered IM with a needle and syringe at a dosage of 45 mcg) as compared to a control group that receives placebo (PBS by Biojector) prime followed at week 36 by the 2012/13 seasonal influenza TIV.

3.2 SECONDARY OBJECTIVE:

- To evaluate whether subjects receiving a DNA prime-TIV boost schedule have a greater frequency of HAI titers that are ≥1:40 or 4-fold greater than baseline compared to those who receive the seasonal influenza TIV alone as assessed at Week 40 (4 weeks after the seasonal influenza TIV injection).

- To evaluate whether subjects receiving a DNA prime-TIV boost schedule have a greater frequency of strain specific H1, H3 and B neutralizing antibodies that are 4-fold greater than baseline compared to those who received the seasonal influenza TIV alone as assessed at Week 40 (4 weeks after the seasonal influenza TIV injection).

3.3 EXPLORATORY OBJECTIVES:

- To evaluate the presence of anti-stem antibodies pre and post vaccination in subjects receiving either regimen, as assessed at Week 40 (4 weeks after the seasonal influenza TIV injection).
- To evaluate other differences in antibody or T cell responses among the two groups of subjects.

4 STUDY DESIGN

This is a Phase 1b, randomized, double-blind study in healthy younger (18-50 years) and older (51-70 years) adults to evaluate the safety, tolerability, and immunogenicity of a prime-boost vaccination regimen with an investigational plasmid DNA vaccine directed towards the 2011/12 influenza vaccine strains as a prime followed 36 weeks later by the 2012/13 influenza trivalent inactivated vaccine (TIV) boost as compared to placebo prime followed 36 weeks later by the 2012/13 seasonal TIV. The hypothesis is that the DNA vaccine will be safe for human

administration and that the prime-boost schedule will elicit a higher titer antibody response than the seasonal TIV alone. The study schema is shown in **Table 4-1** below:

Table 4-1. Study schema

**Protocol VRC 701**			**Injection Schedule**	
**Group**	**Subjects Enrolled**	**Blinded Randomization**	**Day 0**	**Week 36 (±4 weeks)**
Group 1<br>18-50 years	60	Group 1A: n=30	DNA IM Biojector	TIV IM Needle
		Group 1B: n=30	PBS IM Biojector	TIV IM Needle
Group 2<br>51-70 years	60	Group 2A: n=30	DNA IM Biojector	TIV IM Needle
		Group 2B: n=30	PBS IM Biojector	TIV IM Needle
TOTAL	120 subjects		Day 0 injection is blinded;<br>TIV vaccination is open-label	

The expected duration of time on study per subject is approximately 60 weeks.

4.1 Study Population

The study is designed for the enrollment of healthy adults. The following eligibility criteria will be used.

4.1.1 Inclusion Criteria

A subject must meet all of the following criteria:

1. 18 to 70 years old

2. Available for clinical follow-up through Study Week 60

3. Able and willing to complete the informed consent process

4. Willing to donate blood for sample storage to be used for future research

5. Physical examination and laboratory results without clinically significant findings and a Body Mass Index (BMI) <40 within the 70 days prior to enrollment

6. Has received the 2011/2012 licensed influenza vaccine 8 or more weeks prior to enrollment and agrees to receive the 2012/2013 TIV as part of study participation

Laboratory Criteria within 70 days prior to enrollment:

7. Hemoglobin within institutional normal limits

8. White blood cells either within institutional normal range or accompanied by site physician approval as consistent with healthy adult status

9. Platelets = 125,000 – 500,000/mm^3

10. Alanine aminotransferase (ALT) ≤ 2.5 x upper limit of normal (ULN)

11. Serum creatinine ≤ 1 x ULN based on site institutional normal range

Criteria applicable to women of childbearing potential:

12. Negative human chorionic gonadotropin (β-HCG) pregnancy test (urine or serum) on day of enrollment

13. Agree to use an effective means of birth control from 21 days prior to enrollment through 12 weeks after the first study vaccination

4.1.2 Exclusion Criteria

A subject will be excluded if one or more of the following conditions apply:

Women Specific:

1. Breast-feeding or planning to become pregnant during the 12 weeks after enrollment in the study

Subject has received any of the following substances:

2. More than 10 days of systemic immunosuppressive medications or cytotoxic medications within the 12 weeks prior to enrollment or any within the 14 days prior to enrollment

3. Blood products within 16 weeks prior to enrollment

4. Immunoglobulin within 8 weeks prior to enrollment

5. Investigational research agents within 28 days (4 weeks) prior to enrollment

6. Allergy treatment with antigen injections, unless on maintenance schedule and allergy shots could be staggered with the study vaccinations, within 14 days (2 weeks) prior to enrollment

7. Current anti-TB prophylaxis or therapy

Subject has a history of any of the following clinically significant conditions:

8. Contraindication to receiving an FDA-approved seasonal influenza vaccination

9. Serious reactions to vaccines that preclude receipt of study vaccinations, as determined by the site investigator

10. Hereditary angioedema (HAE), acquired angioedema (AAE), or idiopathic forms of angioedema

11. Asthma that is severe, unstable or required emergent care, urgent care, hospitalization or intubation during the previous two years or that is expected to require the use of oral, intravenous or high dose inhaled corticosteroids

12. Diabetes mellitus type I

13. Thyroid disease that is not well-controlled

14. Generalized idiopathic urticaria within the 1 year prior to enrollment

15. Hypertension that is not well controlled

16. Bleeding disorder diagnosed by a doctor (e.g. factor deficiency, coagulopathy, or platelet disorder requiring special precautions), or significant bruising or bleeding difficulties with IM injections or blood draws, or use of blood thinners such as Coumadin or Plavix®.

17. Malignancy that is active or treated malignancy for which there is not *reasonable* assurance of sustained cure or malignancy that is likely to recur during the period of the study

18. Seizure disorder other than: 1) febrile seizures, 2) seizures secondary to alcohol withdrawal more than 3 years ago, or 3) seizures for which no treatment has been required within the 3 years prior to enrollment

19. Asplenia, functional asplenia or any condition resulting in the absence or removal of the spleen

20. Guillain-Barré Syndrome

21. Psychiatric condition that precludes compliance with the protocol; past or present psychoses; disorder requiring lithium; or within 5 years prior to enrollment, a history of suicide plan or attempt

22. Any medical, psychiatric, or other condition that, in the judgment of the investigator, is a contraindication to protocol participation or impairs ability to give informed consent

4.2 STUDY SCHEDULE

The study schedule is presented in the form of a Table in **Appendix III**.

During or following any visit, if there is any concern about the well-being of the subject, the clinical study site will conduct appropriate medical evaluations by history, physical, laboratory or other indicated testing.

4.2.1 Pre-enrollment

Potential study subjects who verbally agree to discuss their medical history may complete a scripted interview questionnaire by telephone or in person that covers protocol inclusion and exclusion criteria that are based on key self-reported history information to identify potential study volunteers.

4.2.2 Screening Visit(s)

Screening for this study may be completed through a general screening protocol after signing consent to be screened or screening consent may be incorporated into a VRC 701-specific consent form. A screening segment for the study will be included in data collection to provide information on reasons for non-enrollment to begin study vaccinations. Evaluations will be done according to eligibility criteria and clinical assessment at screening. Screening evaluations for specific eligibility criteria must be completed within the time interval specified prior to enrollment, but may be repeated as needed to confirm eligibility. The 2011/12 seasonal influenza vaccine may be administered by the site 8 or more weeks prior to an enrollment as part of the screening process to volunteers who have not yet received it. Storage samples of peripheral blood mononuclear cells (PBMCs) and serum collected during screening may be used for assay validation and site proficiency testing. As part of the screening process, an Assessment of Understanding (AoU) should be completed and incorrect answers will be explained to the subject.

4.2.3 Vaccination Enrollment Visit

VRC 701 enrollment is defined as the day of first study injection and is designated Study Day 0. Pregnancy test results for women of reproductive potential must be confirmed as negative on the day of enrollment prior to the study injection. Day 0 evaluations prior to the first injection are the baseline for subsequent safety assessments, except that for any evaluation not performed on Day 0, the baseline will be the screening evaluation.

4.2.4 Administration of Injections:

All Day 0 vaccinations will be administered in a blinded manner according to the randomization assignment. Neither the clinic staff nor the subjects will know whether the vaccine is the DNA vaccine (VRC-FLUDNA061-00-VP at 4 mg dosage) or the phosphate-buffered saline (PBS) placebo. Injections will be administered into deltoid muscle in a 1 mL volume using a Biojector 2000® needle-free injection system (Biojector; Bioject Medical Technologies Inc., Portland, OR). The Biojector will be used as directed by the manufacturer. The Biojector uses sterile, single-use syringes for administration of volume up to 1 mL. The study agent is delivered under pressure by a compressed CO_2 gas cartridge that is stored inside the Biojector. Neither the material being injected nor injection site skin preparation requires deviation from standard procedures. The CO_2 does not come in contact with the injectate and the syringe design prevents any back splatter or contamination of the device by tissue from the subject.

The second injection in all cases will be the 2012/13 seasonal influenza TIV. The TIV injections will be administered IM at the standard dosage of 45 mcg in a 0.5 mL volume into deltoid muscle by needle and syringe, according to the manufacturer's package insert directions.
It is recommended, but not required, that study injections be administered into the non-dominant arm. When choosing an arm for the injection, clinicians should consider whether there is an arm

injury, local skin problem or significant tattoo that precludes administering the injection or will interfere with evaluating the arm after injection.

Following study injection, subjects will be observed for a minimum of 30 minutes after the DNA vaccine injection, and for a minimum of 15 minutes after the TIV injection. Blood pressure and pulse will be taken between 15 and 60 minutes post-injection. The injection site will be inspected for evidence of local reaction. Acute medical care will be provided to subjects for any immediate allergic reactions or other injury resulting from participation in this research study.

7-Day Diary Card and Follow-up: Subjects will be given a "Diary Card", to use as a memory aid, on which to record temperature and symptoms daily for 7 days following each injection. The site may use the written (paper) diary card as a source document or clinician notes obtained by telephone interview as the source of reactogenicity information recorded in the study database. The solicited signs and symptoms on the diary card will include the parameters: unusually tired/feeling unwell, muscles aches (other than at injection site), headache, chills, nausea, and pain/tenderness at injection site. Subjects will also record the day's highest measured temperature and measurement of largest diameter for redness and swelling at injection site.

Subjects will be asked to contact the clinic if they have any concerning signs or symptoms. A clinic visit will be scheduled, if indicated, for the following: rash, urticaria, fever of 38.5˚C (Grade 2) or higher lasting greater than 24 hours, or significant impairment in the activities of daily living (ADL). Additionally, other clinical concerns may prompt a study visit based on the judgment of a study clinician.

4.2.5 Study Visits and Sample Collection

The schedule of follow-up visits, permitted windows for completing the visits, and evaluations performed at each visit is shown in the table in **Appendix III**. After Day 0, deviations from the visit windows in completing study visits are discouraged and will be recorded as protocol deviations, but are permitted, at the discretion of the PI (or designee) in the interest of obtaining subject safety and immunogenicity evaluations following exposure to the investigational vaccine.

At intervals throughout the study, subjects will have blood drawn for safety and immunologic assays.

4.3 CONCOMITANT MEDICATIONS AND PROCEDURES

Current concomitant medications are recorded in the study database at enrollment. Concomitant medications will be updated in the study database if there is an occurrence of an adverse event that requires expedited reporting. Treatment for influenza with antiviral drugs will be recorded on a Influenza Endpoint Case Report Form. Otherwise, a record of concomitant medication changes throughout the study will not be recorded in the study database.

4.4 CRITERIA FOR DISCONTINUING SUBJECT PARTICIPATION

In general, subjects who receive the Day 0 study injection will continue to be followed according to the schedule of safety and immunogenicity evaluations. The second study injection is a licensed seasonal influenza TIV. Therefore, this study does not require discontinuation of the

injection schedule unless circumstances have arisen that constitute a contraindication to administering a licensed TIV injection. Pregnancy is not a contraindication to the TIV, but research blood draws will be adjusted as indicated in Appendix III. A subject may be discontinued from protocol participation for the following reasons:

1. Subject decides to discontinue participation.
2. Subject develops a medical condition that is a contraindication to continuing study participation.
3. The Sponsor or regulatory authority stops the protocol.
4. The Site Investigator of Record (IoR) assesses that it is not in the best interest of the subject to continue participation in the study or that the subject’s compliance with the study is not sufficient.

4.5 CRITERIA FOR PAUSING THE STUDY

The Protocol Chair and UNI-CPSC Medical Monitor will closely monitor and analyze study data as they become available. The VRC Medical Officer will provide an independent review of adverse events on a regular basis. The administration of study injections and new enrollments will be paused if one (or more) subject experiences a Grade 4 or Grade 5 adverse event that is assessed as related to a study vaccine.

The study injections and enrollments would resume only if review of the adverse events that caused the pause resulted in a recommendation to permit further study injections and study enrollments. The Protocol Safety Review Team (PSRT) will conduct the review and make a decision to resume or close the study. Changes in study status will be communicated to the study sites promptly by the UNI-CPSC.

5 SAFETY AND ADVERSE EVENTS

5.1 ADVERSE EVENTS

5.1.1 Adverse Event (AE) Definition

An AE is any untoward or unfavorable medical occurrence in a human subject, including any abnormal sign (e.g., abnormal physical exam or laboratory finding), symptom, or disease temporally associated with the use of study treatment, whether or not considered related to the study treatment.

5.1.2 Adverse Event Reporting in the Study Database

The following guidelines will be used to determine whether or not an adverse event is recorded in the study database.

Each adverse event will be graded according to the table for grading severity of adverse events (see **Appendix IV**).

Solicited adverse events will be recorded in the study database separately with data collection for 7 days after both the first vaccination and the TIV injection as detailed in Section 4.2.4; without the collection of attribution assessments. All unsolicited AEs will be recorded in the study database from receipt of first study injection through 28 days after each study injection. At other

time periods between injections and after the boost, only SAEs (as detailed in **Section 5.2**), new chronic medical conditions, and influenza or influenza-like illness will be recorded through the last study visit. However, cases of influenza or influenza-like illness will be recorded on an influenza endpoints form rather than on an adverse events form.

Any adverse events associated with TIV that meet the criteria for reporting under the Vaccine Adverse Events Reporting System (VAERS) system, are the responsibility of the IoR to report in accordance with the guidance on reportable events available at the website http://vaers.hhs.gov/professionals/index). Refer to the brochure with description and guidance to the VAERS system, which is co-sponsored by the Centers for Disease Control and Prevention (CDC) and the Food and Drug Administration (FDA): http://vaers.hhs.gov/resources/VAERS_Brochure.pdf.

5.2 SERIOUS ADVERSE EVENTS

5.2.1 Serious Adverse Event Definition

The term "Serious Adverse Event" (SAE) is defined in the 21 CFR 312.32 in terms of the following outcomes: Death, a life-threatening adverse event, inpatient hospitalization or prolongation of existing hospitalization, a persistent or significant incapacity or substantial disruption of the ability to conduct normal life functions, or a congenital anomaly/birth defect. Important medical events that may not result in death, be life-threatening, or require hospitalization may be considered serious when, based upon appropriate medical judgment, they may jeopardize the patient or subject and may require medical or surgical intervention to prevent one of the outcomes listed in this definition. Examples of such medical events include allergic bronchospasm requiring intensive treatment in an emergency room or at home, blood dyscrasias or convulsions that do not result in inpatient hospitalization, or the development of drug dependency or drug abuse."

An SAE will be considered "life-threatening" if, in the view of either the investigator or sponsor, its occurrence places the patient or subject at immediate risk of death. It does not include an adverse event or suspected adverse reaction that, had it occurred in a more severe form, might have caused death.

5.2.2 Reporting Serious Adverse Events to the IND Sponsor

Adverse events that meet Serious Adverse Event (SAE) Reporting Requirements must be reported and submitted by the clinical site on an expedited basis to the IND sponsor, VRC/NIAID/NIH, according to sponsor guidelines as follows:

- death
- life-threatening
- results in persistent or significant disability/incapacity
- requires unplanned inpatient hospitalization or prolongation of existing hospitalization
- is a congenital anomaly/birth defect in the offspring of a study subject.
- is an important medical event that may jeopardize the patient or may require intervention to prevent one of the other outcomes listed above.

In addition, any event, regardless of severity, which in the judgment of a site investigator represents a serious adverse event, may be reported on an expedited basis.

A site investigator will communicate an initial SAE report within 24 hours of site awareness of occurrence to VRC (IND Sponsor) through the communication methods provided by the Data Coordinating Center, EMMES Corporation (Rockville, MD).

Any SAE entered into the study database will generate automatic email notification to the UNI-CPSC Medical Monitor and VRC Medical Officer. This or a written report by the study site sent to the attention of the UNI-CPSC Medical Monitor (Email: uniflu@emmes.com or Fax: 301-576-3558) must be submitted within 3 working days in order for the sponsor to comply with regulations mandating sponsor notification of specified SAEs to the FDA within 7 calendar days.

The investigator must submit additional information as it becomes available.

5.2.3 IND Sponsor Reporting to the FDA

It is the responsibility of the IND Sponsor to make the determination of which SAEs are "serious and unexpected suspected adverse reactions" (SUSARs) as defined in 21 CFR 312.32.

- *Suspected adverse reaction* means any adverse event for which there is a reasonable possibility that the drug caused the adverse event.
- *Unexpected Adverse Event* means an AE that is not listed in the Investigator's Brochure or is not listed at the specificity or severity that has been observed.

All SUSARs, as determined by the IND Sponsor, will be reported to FDA as IND Safety Reports and IND Safety Reports will be provided to all participating Investigators by the UNI-CPSC.

The IND Sponsor will also submit an IND Annual Report of the progress of the investigation to the FDA as defined in 21 CFR 312.33.

5.3 REPORTING TO SITE IRBS

Each site IoR is responsible for reporting adverse events to the site IRB in accordance with their IRB's requirements for expedited reporting and continuing review reporting.

Site-specific data reports will be made available to facilitate this continuing review reporting. If there is an IND Safety Report, these will be provided to all sites with instruction as to whether or not any actions need to be taken, such as amendment of consent. Investigators must maintain documentation of compliance with actions required for IND safety reports.

5.4 DATA AND SAFETY MONITORING BOARD

The Protocol Safety Review Team (PSRT) (see **Section 8.8**), will have the primary responsibility for the real-time oversight of safety data, SAE reviews and study pause reviews. The NIAID Intramural Data and Safety Monitoring Board (DSMB) will review cumulative study data twice per year to evaluate safety, study conduct, and scientific validity and integrity of the trial. As part of this responsibility, DSMB members must be satisfied that the timeliness, completeness, and accuracy of the data submitted to them for review are sufficient for evaluation of the safety and welfare of study participants. The DSMB Executive Secretary will be provided with a sealed copy of the randomization codes needed for the DSMB review of the safety data. The DSMB will also assess the performance of overall study operations and any other relevant issues, as

necessary. Following each review, the DSMB will provide its recommendations to the study sponsor, including whether the study should continue without change, be modified, or be terminated.

6 STATISTICAL CONSIDERATIONS AND SAMPLE ANALYSIS

6.1 OVERVIEW

This study is a multi-center trial to assess the safety and tolerability of a prime-boost schedule that includes the investigational recombinant DNA vaccine VRC-FLUDNA061-00-VP (administered IM with a Biojector at a dosage of 4mg), followed at week 36 by the licensed 2012/13 seasonal influenza TIV as compared to a control group that receives placebo (PBS by Biojector) prime followed at week 36 by the 2012/13 seasonal influenza TIV. A preliminary assessment of immunogenicity will also be performed.

6.2 OBJECTIVES

The primary objective relates to safety of the DNA vaccine. The secondary objectives concern immunogenicity endpoints at 4 weeks after the TIV boost (Study Week 40).

6.3 ENDPOINTS

6.3.1 Primary Endpoints: Safety

Assessment of product safety will include clinical observation and monitoring of hematological and chemical parameters. Safety will be closely monitored after injection and evaluated by clinical visits through Study Week 60.The following safety endpoints will be assessed for Study Groups:

- Occurrence of solicited local reactogenicity signs and symptoms for 7 days following each vaccination
- Occurrence of solicited systemic reactogenicity signs and symptoms for 7 days following each vaccination
- Mean change from baseline for safety laboratory measures at intervals through administration of the TIV
- Adverse events of all severities through 28 days after the prime injection and through 28 days after TIV boost
- Serious adverse events through last study visit
- Influenza or influenza-like illness events through last study visit

6.3.2 Secondary Endpoints: Immune Responses

- Proportion of subjects with either a baseline (Day 0) HAI titer < 1:10 and a Week 40 HAI titer ≥ 1:40 or a baseline (Day 0) HAI titer ≥ 1:10 and a minimum four-fold rise from baseline in HAI antibody titer at Week 40.
- Proportion of subjects with a four-fold or greater rise from baseline (Day 0) in specific H1, H3 and B neutralizing antibodies at Week 40.

6.3.3 Exploratory Endpoints: Immune Responses

- Proportion of subjects with either a baseline (Day 0) HAI titer < 1:10 and a post vaccination HAI titer ≥ 1:40 or a baseline (Day 0) HAI titer ≥ 1:10 and a minimum four-fold rise from baseline in post-vaccination HAI titer at Weeks 4, 24, 36 and 60
- Proportion of subjects with a four-fold or greater rise from baseline (Day 0) in specific H1, H3 and B neutralizing antibodies at Weeks 4, 24, 36 and 60
- Proportion of subjects with anti-stem antibodies at Week 40
- Proportion of subjects with positive HA-specific T-cell responses (as measured by ICS assay and ELISpot assay) at Week 40.

6.4 SAMPLE SIZE AND ACCRUAL

The study design is to enroll 120 healthy adults divided equally between two age groups (18-50 years or 51-70 years). Randomization will be stratified by age group with equal allocation to two vaccination schedules. On Day 0, 60 subjects (30 in each age stratum) will receive the investigational DNA vaccine, VRC-FLUDNA061-00-VP, and 60 subjects (30 in each age stratum) will receive a placebo injection. All subjects will receive licensed TIV vaccine at Week 36. The enrollment plan does not include provision for replacing subjects with incomplete vaccination or visit schedules.

Enrollments may occur rapidly at more than one site. The EMMES Corporation will carefully monitor study enrollment and notify all sites and the VRC Protocol Chair when the completion of enrollment is near in order to end recruitment of that age group and to plan how to fairly accommodate, to the degree possible, the enrollment of eligible volunteers that have already been recruited. Enrollment of up to 6 subjects per age group over target accrual is permitted to accommodate screened volunteers and may also compensate for potential loss to follow up over the long time course of the study. The decision to stop study enrollments will be made by the VRC Protocol Chair.

6.4.1 Power Calculations for Evaluation of Safety

The goal of the safety evaluation for this study is to identify safety concerns associated with injections of the investigational vaccine. Primary sample size calculations for safety are expressed in terms of the ability to detect safety or reactogenicity events within each age and vaccination schedule subgroup (n=30). Other sample size calculations for comparing safety rates subgroups are similar to the calculations for immunogenicity shown in Section 6.4.2

The ability of the study to identify safety events will be expressed in terms of the probability of observing a certain number of serious adverse events. Useful values are the minimum true event rate such that the probability of observing at least one event is at least 90% and the maximum true event rate such that the probability of not observing any event is at least 90%. Within each subgroup, there is over 90% chance to observe at least 1 event if the true rate is at least 0.074 and over 90% chance to observe no events if the true rate is less than 0.005

Probabilities of observing no events or more than 1 event within each subgroup are presented in **Table 6-1** for a range of possible true event rates. These calculations provide a more complete picture of the sensitivity of this study design to identify potential safety concerns with the vaccine.

Table 6-1: Probability of Observing Events for Different Safety and Immunogenicity Scenarios within a Subgroup (n=30)

True Event Rate	Pr (observing 0 events)	Pr (more than 1 event)
0.005	0.86	0.01
0.01	0.74	0.04
0.03	0.40	0.23
0.05	0.21	0.45
0.1	0.04	0.82
0.2	0.00	0.99

Table 6-2 gives the upper and lower bounds for 95% exact (Clopper-Pearson) binomial confidence intervals of the true event rates at all possible numbers of events within each subgroup (n=30). If none of the 30 vaccinees experience the event of interest, the 95% exact 2-sided upper confidence bound for the event rate is 0.116.

Table 6-2: 95% Confidence Intervals for the True Rate at All Possible Observed Rates within a Subgroup (n=30)

Observed		95% Confidence Interval	
Rate	Proportion	Lower bound	Upper bound
0/30	0.000	0.000	0.116
1/30	0.033	0.001	0.172
2/30	0.067	0.008	0.221
3/30	0.100	0.021	0.265
4/30	0.133	0.037	0.307
5/30	0.167	0.056	0.347
6/30	0.200	0.077	0.385
7/30	0.233	0.099	0.423
8/30	0.267	0.123	0.459
9/30	0.300	0.147	0.494
10/30	0.333	0.173	0.528
11/30	0.367	0.199	0.561
12/30	0.400	0.226	0.594
13/30	0.433	0.254	0.625
14/30	0.467	0.283	0.656
15/30	0.500	0.313	0.687
16/30	0.533	0.343	0.716
17/30	0.567	0.374	0.745
18/30	0.600	0.405	0.773
19/30	0.633	0.438	0.800
20/30	0.667	0.471	0.827
21/30	0.700	0.505	0.852

Observed		95% Confidence Interval	
Rate	Proportion	Lower bound	Upper bound
22/30	0.733	0.540	0.877
23/30	0.767	0.576	0.900
24/30	0.800	0.613	0.922
25/30	0.833	0.652	0.943
26/30	0.867	0.692	0.962
27/30	0.900	0.734	0.978
28/30	0.933	0.778	0.992
29/30	0.967	0.826	0.999
30/30	1.000	0.882	1.000

6.4.2 Power Calculations for Evaluation of Immune Responses

Table 6-1 gives the probabilities of observing no subjects with immune response or at least 2 subjects with immune responses over a range of underlying response rates. For example, if the true response rate at a particular time point is 0.10, then there is a probability of 0.96 to observe at least one response and a probability of 0.82 to observe at least two responses among the 30 vaccinees.

Table 6-2 is applicable to the immunogenic response rates, and gives the exact 95% confidence intervals over the range of true response rates with a sample size 30 subjects. For example, if we observe 6 responses among the 30 vaccinees, the 95% exact binomial confidence interval of the response rate will range from 0.777 to 0.385.

6.4.3 Power Calculations for Immunogenicity Comparisons

The secondary objectives are to compare the rates of immune response, in terms of positive HA-specific antibody response or strain specific neutralizing antibody, between the two vaccination regimens, the investigational DNA vaccine VRC-FLUDNA061-00-VP followed by the licensed TIV influenza vaccine or the TIV influenza vaccine alone, within each age subgroup (n = 30).

Table 6-3 gives the power of Fisher exact test to compare the two regimens over a range of possible response rates, within each age subgroup. **Table 6-4** presents the minimum difference in response rates that can be detected with 80% power and 30 subjects in each subgroup.

Table 6-3: Power (%) to Detect Difference in Response Rates between Two Subgroups by Fisher's Exact Test

		Group 2 (n=30) Response Rate			
		0.2	0.4	0.6	0.8
Group 1 (n=30) response rate	0.1	10	71	99	99
	0.3	9	8	56	97
	0.5	60	8	8	60
	0.7	97	56	8	9

Table 6-4: Range of response rates in a subgroup and required response rate in the comparison group to have 80% power for a two-sided Fisher Exact Test with type one error = 5%.

Response Rate		
Group 1 (n = 30)	Group 2 (n = 30)	Difference
0.1	0.44	0.34
0.2	0.58	0.38
0.3	0.68	0.38
0.4	0.78	0.38
0.5	0.86	0.36
0.6	0.92	0.32
0.7	0.98	0.28

The exploratory objectives are to compare the proportion of subjects with positive anti-stem antibody or T cell under either the vaccination regimen of the investigational DNA vaccine followed by the licensed TIV or the TIV alone, within each age subgroup. The power calculations presented in Tables 6-3 and 6-4 are applicable to these comparisons.

6.5 STATISTICAL ANALYSIS

Study enrollment is defined in this protocol as being randomized and receiving the first study vaccination. All enrolled subjects will receive at least one vaccination and therefore will provide some safety data, and be included in the Intent-to-treat (ITT) safety analysis.

All statistical analyses will be performed using Statistical Analysis System (SAS), R, or S-Plus statistical software.

No formal multiple comparison adjustments will be employed for safety endpoints or secondary endpoints.

6.5.1 Analysis Variables

The analysis variables consist of baseline, safety, reactogenicity, and immunogenicity variables for primary and secondary objective analyses.

6.5.2 Baseline Demographics

Baseline characteristics including demographics and laboratory measurements will be summarized using descriptive statistics.

6.5.3 Safety Analysis

Reactogenicity: The number and percentage of subjects experiencing each type of reactogenicity sign or symptom will be tabulated by severity. For a given sign or symptom, each subject's reactogenicity will be counted once under the maximum severity for all assessments.

Adverse Events: Adverse Events (AEs) will be coded into Medical Dictionary for Regulatory Activities (MedDRA) preferred terms. The number and percentage of subjects experiencing each specific adverse event will be tabulated by severity and relationship to treatment. For the calculations in these tables, each subject's adverse event will be counted once under the maximum severity or strongest recorded causal relationship to treatment.

A complete listing of AEs for each subject will provide details including severity, relationship to treatment, onset, duration and outcome.

Safety Laboratory Values: Safety laboratory values will be summarized as the mean change from baseline along with 95% confidence interval at each timepoint measured in the study. Boxplots of safety laboratory values will be generated for baseline values and for values measured during the course of the study. Each boxplot will show the 1st quartile, the median, and the 3rd quartile, with values smaller than the 1st quartile or larger than the 3rd quartiles plotted as outliers. If appropriate, horizontal lines representing boundaries for abnormal values will be plotted.

6.5.4 Analysis of Immune Responses

The statistical analysis for immunogenicity will employ the intent-to-treat principle, i.e., all data from enrolled subjects will be used. In the final analysis of immunogenicity, if there are cases of a subject receiving a regimen different from the assignment, then an as-treated analysis will be performed.

If assay data are qualitative (i.e., positive or negative) then analyses will be performed by tabulating the frequency of positive response for each assay at each time point that an assessment is performed. Binomial response rates will be presented with their corresponding exact 95% confidence interval estimates. Fisher's exact tests will be used to compare any two vaccine groups to each other. Missing responses will be assumed to be missing at random, i.e., conditional on the observed data the missingness is independent of the unobserved responses.

Graphical descriptions of the longitudinal immune responses will also be given.
Some immunologic assays have underlying continuous or count-type readout that is often dichotomized into responder/nonresponder categories. For these assays, graphical and tabular summaries of the underlying distributions will be made. These summaries may be performed on transformed data (e.g., log transformation) to better satisfy assumptions of symmetry and homoscedasticity.

6.5.5 Interim Analyses

Independent Safety Reviews: The protocol safety review team will review safety data routinely throughout the study. The study will utilize both electronic database features and reviews by designated safety review personnel to identify in a timely manner if any of the safety pause rules of the study are met. The NIAID Intramural DSMB will provide an independent safety review at scheduled intervals to coincide with their biannual meeting schedule.

Immunogenicity Review: One interim analysis of immunogenicity may be performed when the HA HAI assays at 4 weeks after the TIV injection have been completed for the study. Reports

providing results by schedule, without identifying results by individual study I.D., will be provided to VRC solely for the purpose of informing decisions related to future trials in a timely manner. The results should in no way influence the conduct of the VRC 701 trial in terms of early termination or later safety or immunogenicity endpoint assessments or unblind the study sponsor, clinical site staff, laboratory staff or subjects to individual subject's treatment assignments. Interim analyses of ICS and other immunogenicity assays may also be performed.

6.5.6 Randomization of Treatment Assignments and Unblinding Criteria

Randomizations will be done online using the enrollment module of The EMMES Corporations Internet Data Entry System (IDES). The randomization code will be prepared by statisticians at The EMMES Corporation and included in the enrollment module for the trial. The randomization code will link to the treatment assignment. To decrease the potential for subject dropouts during the period between randomization and initial vaccination, the electronic data system will assign each subject a randomization code after the eligibility to begin Day 0 study injections has been entered into the system.

For each clinical site, randomization will be stratified by age (18-50 or 51-70), with 1:1 allocation within each stratum to either recombinant DNA vaccine VRC-FLUDNA061-00-VP followed by 2012/13 seasonal influenza TIV at Week 36 -- or -- PBS placebo followed by 2012/13 seasonal influenza TIV at Week 36. Manual back-up procedures and instructions will be provided for use in the event that the site temporarily loses access to the Internet or the online enrollment system is unavailable to a study site.

Screening records will be kept to document the reason why an individual was screened but not enrolled into the clinical trial.

The vaccinations will be prepared by an unblinded site pharmacist or otherwise qualified personnel who will not be involved in any subject assessments and who will not discuss randomizations with study clinicians. The subjects, the study personnel who perform study vaccinations and assessments, data entry personnel at the sites, and laboratory personnel performing immunologic assays will be blinded to the treatment assignment of the first injection (DNA vaccine or placebo). Subjects and all study personnel will know that all subjects are scheduled to receive the licensed influenza vaccine for the second injection. The DSMB may receive data in aggregate and presented by treatment group, but without the treatment group identified. The DSMB may be unblinded to individual study treatment assignments, as needed, to adequately assess safety issues.

If necessary, the IoR (or designee), Protocol Chair, and UNI CPSC Medical Monitor may agree that management of an adverse event requires emergency unblinding of an individual subject's assignment following the first injection. A designated individual at each site will be provided with a code list for emergency unblinding purposes, which will be kept in a secure place. This will be documented as a protocol deviation and the Protocol Statistician, the site IRB, and the DSMB will be notified that an early unblinding has occurred and provided with a statement explaining the medical necessity for the early unblinding.

7 PHARMACY AND VACCINE ADMINISTRATION PROCEDURES

The study groups and vaccination schedules are shown in **Table 4.1**. Refer to **Section 2** for information about manufacturing of study agents.

7.1 STUDY AGENTS

An investigational study agent may only be shipped to the site pharmacy when the protocol is IRB-approved at the study site. This study includes one investigational vaccine, one placebo for the investigational vaccine, and one non-investigational seasonal TIV vaccine as follows:

- VRC-FLUDNA061-00-VP 4 mg/mL (DNA vaccine)
- Phosphate buffered saline (PBS) as placebo control for the DNA vaccine
- 2012-2013 Seasonal Influenza TIV

The TIV vaccine used for the week 36 injection will be from the 2012-2013 season and will be from a commercially available vaccine prepared for the Northern Hemisphere (NH).

7.2 STUDY AGENT PRESENTATION AND STORAGE

7.2.1 Study Agent Labels

At the time of delivery of the study agent to the pharmacy, the labels for study agent VRC-FLUDNA061-00-VP (DNA vaccine) and PBS placebo will have specific product information (e.g., part number, lot number, fill volume, storage temperature) included on the product vial labels. The labels will contain an Investigational Use Statement ("Caution: New Drug – Limited by Federal Law to Investigational Use") and manufacturer information.

The 2012-2013 Seasonal Influenza TIV will be a commercial, licensed vaccine in its original manufacturer's packaging.

7.2.2 Study Agent Storage

Temperature excursions that are outside of the normal allowance for the storage device in which each type of product is kept will be reported to the study sponsor via the study coordinating center (The EMMES Corporation). The excursion must be evaluated and investigated and action must be taken to restore and maintain the desired temperature limits. The site IoR is ultimately responsible for notification of the sponsor, but may delegate this responsibility to a pharmacist. Pending the outcome of the investigation, the site will be informed if continued clinical use of the product is acceptable.

VRC-FLUDNA061-00-VP: Upon release by VRC/NIAID/NIH, the DNA vaccine vials will be shipped within the recommended temperature range using appropriate shipping configurations, to the study pharmacist, and will be stored until use at -45°C to -10°C in a qualified, continuously monitored, temperature-controlled freezer.

Phosphate Buffered Saline (PBS): PBS, for use as the placebo, is supplied in a 3mL glass vial containing 1.2ml of a clear colorless isotonic sterile solution. The product will be stored until use at -45°C to -10°C in a qualified, continuously monitored, temperature-controlled freezer

2012-2013 TIV: The seasonal influenza TIV will be stored according to the label instructions and released by the pharmacist to the designated clinical staff for administration to study subjects.

7.3 PREPARATION OF STUDY AGENT FOR INJECTION

This section describes how the site pharmacist or designee will prepare the DNA vaccine or placebo and TIV injections. Clinician instructions on how to select an arm and administer the injection are in **Section 4.2.4**.

7.3.1 Preparation of VRC-FLUDNA061-00-VP

The DNA vaccine is supplied as a 2 mL glass vial containing a clear colorless isotonic sterile solution. Each vial contains 20% over the amount to be injected in cGMP grade phosphate-buffered saline. Vials are intended for single use only, and thus do not contain a preservative. They should not be refrozen after thawing. Each vial (4 mg/mL) contains a volume of 1.2 mL (4.8 mg).

Refer to the group assignment for the study subject. For subjects to whom the DNA vaccine is to be administered, remove a vial of the DNA vaccine 4 mg/mL from the freezer. Allow the vial to equilibrate to room temperature (15 to 30º C). Swirl the contents gently. Using aseptic technique, withdraw 1 mL of the DNA vaccine from the vial into the Biojector syringe, remove air bubbles and cap the syringe. The pharmacy will label the syringe prior to delivery to the clinic with the subject identifier and the date and time allowance for administration; the label will not include the product type information.

One 1 mL injection of the 4 mg/mL preparation will be administered for each 4 mg dose of DNA vaccine. A dose of vaccine will be prepared in the pharmacy at the site, and the prepared Biojector syringe labeled with the subject identifier will be delivered to the clinic for administration. The pharmacy/designated site personnel will also label with information about date and time after which the preparation may not be used. The injection must be administered within 4 hours after removing the vial from the freezer.

7.3.2 Preparation of Phosphate Buffered Saline (PBS)

The PBS (placebo) is supplied in 3 mL glass vials, each containing 1.2 mL PBS at pH 7.2. Vials are intended for single use only, and thus do not contain a preservative. They should not be refrozen after thawing.

Refer to the group assignment for the study subject. For subjects to whom the placebo control is to be administered, remove a vial of the PBS from the freezer and thaw at room temperature. Draw up 1 mL of PBS placebo (pH 7.2) into the Biojector syringe. Remove all air bubbles from the syringe and cap it. A dose of placebo will be prepared in the pharmacy and the prepared Biojector syringe labeled with the subject identifier (but not indicating whether vaccine or placebo) will be delivered to the clinic for administration. The pharmacy will label the syringe prior to delivery to the clinic with the subject identifier and the date and time allowance for administration; the label will not include the product type information. The injection must be administered within 4 hours after removing the vial from the freezer.

7.3.3 Preparation of 2012-2013 TIV Injection

Each 0.5 mL dose of the seasonal influenza TIV contains 45 mcg total comprised of 15 mcg of influenza virus hemagglutinin of 3 different strains as approved by the FDA for the 2012-13 influenza season. Each injection is administered in the clinic in accordance with the package insert instructions.

7.4 STUDY AGENT ACCOUNTABILITY

7.4.1 Documentation

Each study site will be responsible for maintaining an accurate record of the codes, inventory, and an accountability record of the investigational vaccine supplies for this study at their site. Electronic documentation as well as paper copies will be used.

7.4.2 Disposition

The empty vials and the unused portion of a vial will be discarded in a biohazard containment bag that will be incinerated or autoclaved. Any unopened vials that remain at the end of the study will be discarded at the discretion of the VRC in accordance with policies that apply to investigational agents. Partially used vials or expired prepared doses cannot be administered to other subjects nor used for *in vitro* experimental studies and will be discarded as indicated above

8 HUMAN SUBJECTS PROTECTION

8.1 INSTITUTIONAL REVIEW BOARD

A copy of the protocol, proposed informed consent and any proposed advertising material will be submitted to the site IRB for review and approval.

The Site IoR will submit and, where necessary, obtain approval from the IRB for subsequent protocol amendments and changes to the informed consent document. The Site IoR is responsible for ensuring proper IRB notification of deviations from the protocol or serious adverse events occurring at the site and other adverse event reports received from the VRC, NIAID, in accordance with the protocol and local IRB policies. The IoR will be responsible for obtaining annual IRB approval/renewal throughout the duration of the protocol. Documentation of the IRB approval and FWA number will be provided for the Sponsor’s records.

8.2 SUBJECT RECRUITMENT AND ENROLLMENT

Subjects for this study will be recruited by the sites in accordance their site IRB standard for recruitment practices. Effort will be made to include women and minorities in proportions similar to that of the community from which they are recruited.

8.2.1 Participation of Children

Healthy children can participate in research studies considered as "not greater than minimal risk" and can participate in research with greater than minimal risk only when it presents the prospect of direct benefit to the individual child or is likely to yield generalizable knowledge about the child's disorder or condition and meets the criteria in 45 CFR 46, Subpart D.

The investigational vaccines in this study have been assessed as eligible for evaluation in children. To accomplish this in a manner most likely to obtain knowledge generalizable to

children, they will be evaluated in a separate protocol designed for children. Therefore children are not eligible for this protocol.

8.3 INFORMED CONSENT

The provided template informed consent (**Appendix I)** will be used to guide development of the site-specific consent forms. Only an IRB-approved consent form will be used to consent subjects for participation in the study. The changes in the informed consent template by the site should be approved with the VRC Program Officer before submission to respective IRB. The written informed consent documents will be prepared in the language(s) of the potential subject population. Before a subject's participation in the protocol, it is the investigator's responsibility to ensure that written informed consent is obtained from the subject after adequate explanation of the aims, methods, anticipated benefits, and potential hazards of the protocol.

The acquisition of informed consent should be documented in the subject's records, as required by 45 CFR 46.117, and the informed consent form should be signed and personally dated by the subject and by the person who conducted the informed consent discussion. An original signed informed consent form should be retained by the site and a signed copy of the consent form should be provided to the subject.

8.4 SUBJECT CONFIDENTIALITY

The investigators at each site must ensure that the subject's anonymity is maintained. Subjects will not be identified in any reports of this study. All records will be kept confidential to the extent provided by federal, state and local law. Medical records will be made available for review when required by authorized agencies and regulatory authorities only under the guidelines set by the Federal Privacy Act. Direct access includes examining, analyzing, verifying, and reproducing any records and reports that are important to the evaluation of the study. The above named representatives will review study-related records without violating the confidentiality of the subjects. Stored study research samples will be labeled by a code (such as a number) that only the site clinical study team can link to the subject. The requirement to maintain subject confidentiality and inform subjects about review of study-related records is included in the study informed consent documents.

8.5 RISKS AND BENEFITS

There is no benefit to the subject for participating in this protocol, but society may benefit from knowledge gained from research on the donated specimens.

8.5.1 <u>Risks of Blood Collections:</u>

The blood collection procedures are common in routine medical practice. The risks of blood sample collection are minimal and consist of mild discomfort at the sample collection site. The procedure may cause mild pain, bruising, fainting, and, rarely, infection at the site where the blood is taken.

8.5.2 <u>Risks of the DNA Vaccine:</u>

This is the first study in humans of the trivalent seasonal HA DNA vaccine, VRC-FLUDNA061-00-VP, although two of the three plasmids have been administered as part of other HA DNA vaccines. The risks noted are based on risks from the earlier DNA vaccine studies of similar vaccines, as well as risks of vaccines in general and results of previous studies with other investigational DNA vaccines.

Subjects may exhibit general signs and symptoms associated with administration of a vaccine injection, including fever, chills, rash, aches and pains, nausea, headache, dizziness and fatigue. These side effects , when they occur, are generally short term, mild to moderate severity, and usually do not require treatment.

Theoretical risks of DNA vaccines include: muscle damage, antibodies to DNA, insertion of the vaccine DNA into genomic DNA (a potential cancer risk), or insertion of the vaccine DNA into a bacteria or virus. Experimental data have not confirmed these theoretical risks.

In previous VRC DNA vaccine studies, placebo and vaccine recipients were noted to have occasional asymptomatic and self-limited changes in the laboratory tests routinely followed during the clinical trial. Urticaria has been reported as an infrequent adverse event possibly related to DNA vaccines.

Investigational DNA vaccines administered via Biojector have been associated with mild skin lesions (0.5-1.0 cm diameter) at the vaccination site. In these cases, a small scab formed within 1-2 weeks after immunization and came off after a few days. The skin healed without treatment within a few weeks. One skin biopsy was obtained on Day 6 post vaccination. It showed subcutaneous and dermal perivascular lymphocytic inflammation. There were rare eosinophils and rare giant cells noted, and the infiltrate was composed entirely of CD3 positive cells. It included both $CD4^+$ and $CD8^+$ T cells. The process appears to be primarily a subcutaneous inflammatory response to vaccination with cutaneous manifestations.

There may be other unknown side effects.

8.5.3 Risks of the Seasonal TIV Vaccine:

Occasionally, adult recipients of seasonal influenza TIV may develop influenza-like reactions such as fever, body aches, headache, malaise, myalgia and/or nausea. These reactions are usually greatest within the first 24 hours after vaccination and last for 1 to 2 days. Some subjects may develop reactions at the site of vaccination (redness, swelling, pain, or tenderness). Analgesics (e.g., ibuprofen and acetaminophen) and rest will generally relieve or moderate these symptoms. These reactions should go away in 1 to 4 days and not require additional treatment.

Acute and potentially life-threatening allergic reactions are also possible. Since the vaccine may contain limited quantity of egg protein, this protein can induce immediate hypersensitivity reactions among person who have severe allergy. Allergic reactions include hives, angioedema, allergic asthma, and systemic anaphylaxis.

During the swine influenza vaccine campaign of 1976, about 1 per 100,000 vaccine recipients developed a paralytic illness called Guillain-Barré Syndrome. This has not been seen consistently with other influenza vaccines. Most persons who develop Guillain-Barré Syndrome recover completely.

There may be other unknown side effects.

8.5.4 Other Risks:

The effect of the investigational DNA vaccine on a fetus or nursing baby is unknown; female subjects of reproductive potential will be required to agree to use birth control for sexual intercourse beginning 21 days prior to enrollment and continue through 12 weeks after first study injection. Women who are pregnant or nursing will be excluded from enrollment into the study.

The licensed seasonal influenza TIV that everyone will get as the second study injection is approved for administration during pregnancy. However, because this is a research study, women of reproductive potential will be asked to notify the site immediately upon learning of a pregnancy during this study and will be tested for pregnancy prior to administration of TIV. The amount of blood drawn will be reduced. The site will be contacted to ask about the outcome of a pregnancy that begins during the study.

It is possible that the standard medical tests performed as part of this research protocol will result in new diagnoses. Depending upon the medical findings and consequences of being provided with the new medical information about health status, the study subject may view this aspect of study participation as either a risk or a benefit. Any such information will be shared and discussed with the subject and, if requested by the subject, will be forwarded to the subject's primary health care provider for further workup and management.

8.5.5 Benefits:

Study participants may have no direct benefit from participation in this study. This protocol is not designed to provide treatment for any condition.

8.6 PLAN FOR USE AND STORAGE OF BIOLOGICAL SAMPLES

To be eligible for this protocol, subjects must be willing to allow stored specimens to be used in the future for studying infectious diseases, immune function, vaccine responses and other medical conditions, and must also be willing to have genetic tests, including HLA typing performed. If tests performed at a study site show evidence of any acute or chronic condition, subjects will be informed of the results and advised to seek appropriate medical care for the condition. In general, testing performed at a research laboratory is not for diagnostic purposes and results will not be available to the study site or study subject.

Intended Use of the Samples/Specimens/Data:

Samples, specimens and data collected under this protocol may be used to study infectious diseases such as influenza, immune function, vaccine responses, genetic factors in immune responses, other medical conditions and for research assay validation.

How Samples, Specimens and Data from Sample Use Will Be Stored:

All of the stored study research samples will be labeled by a code (such as a number) that only the study site can link to the subject. Samples will be stored in secure facilities with controlled access at the sites, a central repository maintained by NIH or at central laboratories associated with the study. Samples collected for research may be transferred for testing to the approved collaborators. Data will be kept secure. Only approved investigators or their designees will have access to samples and data. The NIAID Vaccine Immune T-Cell and Antibody Laboratory (NVITAL) in Gaithersburg, MD, under the direction of the VRC, NIAID, NIH (Bethesda, MD)

and research labs at or contracted to the VRC or The EMMES Corporation will be the involved in conducting assays with stored samples.

In the future, other investigators (both at NIH and outside) may wish to study these samples and/or data. Regulatory approval through the proper human subjects protection agency will be sought prior to any sharing of samples that constitutes human subject research. The research use of stored, unlinked or unidentified samples may be exempt from the need for IRB review and approval. When appropriate, exemption may be obtained through the proper regulatory procedures.

8.7 Compensation

Compensation for study visits and procedures will be provided to offset the time and inconvenience of participation. Subjects will be compensated in accordance with the site-specific IRB approval.

8.8 Safety Monitoring

8.8.1 Protocol Safety Review Team

Each site IoR is responsible for ensuring daily review of the site's clinical safety data as it becomes available. The Protocol Safety Review Team (PSRT) includes the Protocol Chair, IND Sponsor Medical Officer, the UNI-CPSC Medical Monitor and each site IoR or designee. The PSRT will review the summary study safety data reports weekly for the first 4 weeks after study injections begin and then monthly until 4 weeks after all subjects have completed the first study injection in order to be certain that the investigational vaccine has an acceptable safety profile. The PSRT will be notified and convened to review any study pauses. The Protocol Chair, IND Sponsor Medical Officer, the UNI-CPSC Medical Monitor will continue to monitor the cumulative study safety data reports on at least a monthly basis through completion of the last study visit.

8.8.2 DSMB

As described in Section 5.4, the DSMB will review safety data twice per year at their regularly scheduled meetings and will have access to the randomization code.

9 ADMINISTRATION AND LEGAL OBLIGATIONS

9.1 Protocol Initiation, Amendments and Termination

Each site must receive IRB approval and approval of The EMMES Corporation before initiating the study at the site. All amendments will also be submitted to the site IRBs for approval. The VRC, NIAID, NIH reserves the right to terminate the study. Each IoR will notify the respective site IRB of the study termination in writing and provide documentation to The EMMES Corporation.

9.2 Study Documentation and Study Records Retention

The site IoR will maintain a list of appropriately qualified persons to whom trial duties have been delegated. The site IoR is responsible for ensuring that staff maintains a comprehensive and centralized filing system of all study-related (essential) documentation, suitable for inspection at

any time by representatives from the VRC, IRB, The EMMES Corporation and/or applicable regulatory authorities. Elements include but are not limited to:

- Subject files containing completed informed consent forms and supporting copies of source documentation
- Study files containing the protocol with all amendments and copies of all correspondence with the IRB

In addition, all original source documentation must be maintained and readily available.

The EMMES Corporation is responsible for ensuring that records and documents pertaining to the conduct of this study, including CRFs, source documents, consent forms, laboratory test results, and medication inventory records, must be retained by the investigator for at least 2 years following submission of a Biologics License Application or until VRC, NIAID authorizes transfer or destruction of study records. No study records will be destroyed without prior authorization from NIAID.

9.3 DATA COLLECTION AND PROTOCOL MONITORING

9.3.1 Data Capture Methods

Clinical research data will be collected and recorded by the study sites in a timely fashion in a secure electronic web-based clinical data management system (CDMS) provided by The EMMES Corporation as defined by the contract. Immunological testing on collected, coded blood samples may be performed in batches at central laboratories. Extracted data without subject identifiers will be sent to the statisticians for statistical analysis as needed. The final study database and statistical evaluations will be transferred to the VRC, NIAID at the study completion.

9.3.2 Source Documents and Access to Source Data/Documents

Each participating site will maintain appropriate medical and research records for this trial, in compliance with ICH-GCP, regulatory and institutional requirements for the protection of confidentiality of subjects. As part of participating in the NIAID-sponsored study, each site will permit authorized representatives of the VRC, NIAID, and regulatory agencies to examine (and when required by applicable law, to copy) clinical records for the purposes of quality assurance reviews, audits and evaluation of the study safety and progress.

Source data are all information, original records of clinical findings, observations, or other activities in a clinical trial necessary for the reconstruction and evaluation of the trial. Examples of these original documents and data records include, but are not limited to, medical records, laboratory reports, pharmacy records and other research records maintained for the clinical trial.

9.3.3 Protocol Monitoring

The study data integrity and compliance with the protocol will be assured by the monitoring of the study documentation and study conduct at the sites by The EMMES Corporation. Routine data monitoring and protocol compliance will be performed by the site investigators and study coordinator on an ongoing basis. The study clinical monitoring plan and the data quality

monitoring plan will be developed and followed by The EMMES Corporation in consultation with the VRC Program Officer.

9.4 Language

All written information and other material to be used by subjects and investigative staff must use vocabulary and language that are readily understood.

9.5 Policy Regarding Research-Related Injuries

The study site will provide immediate medical care for any injury resulting from participation in this research. In general, the VRC, the NIH, or the Federal Government will not provide long-term medical care or financial compensation for research-related injuries.

10 REFERENCES

1. CDC, *Serum cross-reactive antibody response to a novel influenza A (H1N1) virus after vaccination with seasonal influenza vaccine.* MMWR - Morbidity & Mortality Weekly Report, 2009. **58**(19): p. 521-4.
2. Subbarao, K., B.R. Murphy, and A.S. Fauci, *Development of effective vaccines against pandemic influenza.* Immunity, 2006. **24**(1): p. 5-9.
3. Luke, C. and K. Subbarao, *Vaccines for pandemic influenza.* Emerging Infectious Diseases, 2006. **12**(1): p. 66-72.
4. WHO, *Influenza (Seasonal) Fact Sheet*, 2009: Geneva, Switzerland.
5. Kilbourne, E., *Influenza pandemics of the 20th century.* Emerg Infect Dis [serial on the Internet]. 2006. **available from http://www.cdc.gov/ncidod/EID/vol12no01/05-1254.htm**.
6. WHO. *Statement to the press by WHO Director-General Dr Margaret Chan 11 June 2009* 2009; Available from: http://www.who.int/mediacentre/news/statements/2009/h1n1_pandemic_phase6_20090611/en/index.html
7. WHO. *Recommendations for influenza vaccines*. Available from: http://www.who.int/csr/disease/influenza/vaccinerecommendations/en/index.html.
8. Fiore, A.E., et al., *Prevention and control of seasonal influenza with vaccines: recommendations of the Advisory Committee on Immunization Practices (ACIP), 2009.* MMWR Recomm Rep, 2009. **58**(RR-8): p. 1-52.
9. Targonski, P.V., R.M. Jacobson, and G.A. Poland, *Immunosenescence: role and measurement in influenza vaccine response among the elderly.* Vaccine, 2007. **25**(16): p. 3066-9.
10. Harper, S.A., et al., *Prevention and control of influenza: recommendations of the Advisory Committee on Immunization Practices (ACIP).* MMWR Recomm Rep, 2004. **53**(RR-6): p. 1-40.
11. Novel Swine-Origin Influenza, A.V.I.T., *Emergence of a Novel Swine-Origin Influenza A (H1N1) Virus in Humans.* N Engl J Med, 2009. **360**(25): p. 2605-2615.
12. Du, L., Y. Zhou, and S. Jiang, *Research and development of universal influenza vaccines.* Microbes and Infection, 2010. **12**(4): p. 280-286.
13. Alexander, J., et al., *Identification of broad binding class I HLA supertype epitopes to provide universal coverage of influenza A virus.* Human Immunology, 2010. **71**(5): p. 468-474.
14. Wang, T.T., et al., *Broadly Protective Monoclonal Antibodies against H3 Influenza Viruses following Sequential Immunization with Different Hemagglutinins.* PLoS Pathog, 2010. **6**(2): p. e1000796.
15. Wei, C.-J., et al., *Cross-Neutralization of 1918 and 2009 Influenza Viruses: Role of Glycans in Viral Evolution and Vaccine Design.* Science Translational Medicine, 2010. **2**(24): p. 24ra21.
16. Wei, C.J., et al., *Induction of broadly neutralizing H1N1 influenza antibodies by vaccination.* Science, 2010. **329**(5995): p. 1060-4.
17. Thomas, P.G., et al., *Cell-mediated protection in influenza infection.* Emerging Infectious Diseases, 2006. **12**(1): p. 48-54.
18. Doherty, P.C., *Toward a broadly protective influenza vaccine.* The Journal of Clinical Investigation, 2008. **118**(10): p. 3273-3275.

19. Jameson, J., J. Cruz, and F.A. Ennis, *Human Cytotoxic T-Lymphocyte Repertoire to Influenza A Viruses.* The Journal of Virology, 1998. **72**(11): p. 8682-8689.
20. McElhaney, J.E. and J.P. Dutz, *Better influenza vaccines for older people: what will it take?* J Infect Dis, 2008. **198**(5): p. 632-4.
21. McElhaney, J.E., et al., *T Cell Responses Are Better Correlates of Vaccine Protection in the Elderly.* J Immunol, 2006. **176**(10): p. 6333-6339.
22. Tavel, J.A., et al., *Safety and immunogenicity of a Gag-Pol candidate HIV-1 DNA vaccine administered by a needle-free device in HIV-1-seronegative subjects.* J Acquir Immune Defic Syndr, 2007. **44**(5): p. 601-5.
23. Graham, B.S., et al., *Phase 1 safety and immunogenicity evaluation of a multiclade HIV-1 DNA candidate vaccine.* J Infect Dis, 2006. **194**(12): p. 1650-1660.
24. Catanzaro, A.T., et al., *Phase I clinical evaluation of a six-plasmid multiclade HIV-1 DNA candidate vaccine.* Vaccine, 2007. **25**(20): p. 4085-92.
25. Koup, R.A., et al., *Priming Immunization with DNA Augments Immunogenicity of Recombinant Adenoviral Vectors for Both HIV-1 Specific Antibody and T-Cell Responses.* PLoS One, 2010. **5**(2): p. e9015.
26. Kibuuka, H., et al., *A phase 1/2 study of a multiclade HIV-1 DNA plasmid prime and recombinant adenovirus serotype 5 boost vaccine in HIV-Uninfected East Africans (RV 172).* J Infect Dis, 2010. **201**(4): p. 600-7.
27. Jaoko, W., et al., *Safety and immunogenicity study of Multiclade HIV-1 adenoviral vector vaccine alone or as boost following a multiclade HIV-1 DNA vaccine in Africa.* PLoS One, 2010. **5**(9): p. e12873.
28. Martin, J.E., et al., *A DNA vaccine for Ebola virus is safe and immunogenic in a phase I clinical trial.* Clin Vaccine Immunol, 2006. **13**(11): p. 1267-77.
29. Martin, J.E., et al., *A SARS DNA vaccine induces neutralizing antibody and cellular immune responses in healthy adults in a Phase I clinical trial.* Vaccine, 2008. **26**(50): p. 6338-43.
30. Martin, J.E., et al., *A West Nile virus DNA vaccine induces neutralizing antibody in healthy adults during a phase 1 clinical trial.* J Infect Dis, 2007. **196**(12): p. 1732-40.
31. Ledgerwood, J.E., et al., *A West Nile virus DNA vaccine utilizing a modified promoter induces neutralizing antibody in younger and older healthy adults in a phase I clinical trial.* J Infect Dis, 2011. **203**(10): p. 1396-404.
32. Ledgerwood, J.E., et al., *DNA priming and influenza vaccine immunogenicity: two phase 1 open label randomised clinical trials.* Lancet Infect Dis, 2011. **11**(12): p. 916-924.
33. Graham, B.S., et al. *Evaluation of candidate DNA HIV-1 vaccine delivery by Biojector or needle and syringe in healthy adults (VRC 008).* in *AIDS Vaccine 2007*. 2007. Seattle, WA.
34. Betts, M.R., J.P. Casazza, and R.A. Koup, *Monitoring HIV-specific CD8+ T cell responses by intracellular cytokine production.* Immunol Lett, 2001. **79**(1-2): p. 117-25.
35. Helms, T., et al., *Direct visualization of cytokine-producing recall antigen-specific CD4 memory T cells in healthy individuals and HIV patients.* J Immunol, 2000. **164**(7): p. 3723-32.
36. Barouch, D.H., et al., *A human T-cell leukemia virus type 1 regulatory element enhances the immunogenicity of human immunodeficiency virus type 1 DNA vaccines in mice and nonhuman primates.* J Virol, 2005. **79**(14): p. 8828-34.

37. Yang, Z.Y., et al., *A DNA vaccine induces SARS coronavirus neutralization and protective immunity in mice.* Nature, 2004. **428**(6982): p. 561-4.

Appendix I: Template Informed Consent Forms

The sample informed consent forms are provided to guide development of a site-specific consent form. Only an IRB-approved consent forms will be used to consent subjects for participation in the study.

Study Consent, Template Version 1.0

Template Informed Consent Form for Study Participation

STUDY TITLE: VRC 701: A Double-Blind, Randomized Phase IB Study of the Safety and Immunogenicity of a Prime-Boost Schedule of the 2011/12 Investigational DNA Trivalent Influenza Vaccine, VRC-FLUDNA061-00-VP, Followed by the 2012/13 Seasonal Influenza Trivalent Inactivated Vaccine (TIV) Compared to 2012/13 TIV Alone in Healthy Adults Ages 18-50 and 51-70 Years

INTRODUCTION

We invite you to take part in a research study at the ___________________________.

You are here to decide whether to join the research study described below. The study is sponsored by the National Institutes of Health. It is up to you whether or not you want to be a part of this study. This is no penalty or loss of benefits for choosing to not participate. Please ask questions and discuss this study with anyone you want and take as much time as you need to decide.

[SCREENING (delete this section if site has separate screening protocol or separate screening consent)]

Before you can enroll in the investigational influenza vaccine study, you will be screened for eligibility. You will need to sign this consent form before we can do the screening.

Screening involves a physical exam and blood tests to check your general health status. If you are a woman you will be asked about your health related to the possibility of becoming pregnant, birth control use and tested for pregnancy if applicable. During screening, we may collect some blood to store for research. We will ask you about your general health history and influenza history. We will ask you about medications you are taking and recent vaccinations.

We will review the screening results with you and tell you if the screening results may show you are not eligible to join the study. You cannot be in another research study where you receive a study product and also enroll in this study.

PURPOSE OF THE VACCINE STUDY

This is a research study of two vaccines for seasonal influenza (“flu”). One is an experimental DNA vaccine. The other is the standard licensed vaccine for the prevention of seasonal influenza that is referred in this consent as “trivalent inactivated vaccine” or “TIV”. The experimental DNA vaccine is not approved by the Food and Drug Administration (FDA) for preventing “flu” infection. The main purpose of this study is to see if the experimental DNA vaccine is safe and whether there are any side effects. Another goal is to study blood samples in the laboratory for immune responses and to see if the DNA vaccine results in better immune responses.

You are eligible to participate in this study because:

- you have completed the screening process,
- you are between 18 and 70 years old,
- you have physical exam and blood test results that meet eligibility requirements, and
- you do not have any significant medical problems as determined by your screening.

The study plan is to enroll 120 people in this study at several sites through the US. Study participation will last about 60 weeks for each person. While on the study, you will be monitored for vaccine-related side effects. In this study, the study injections (shots) will be given to people in the upper arms.

STUDY INJECTIONS

Vaccines are substances used to try to create resistance (or immunity) to a disease. You cannot get influenza infection from study injections because none of the injections contain influenza virus. The investigators will study if getting the DNA vaccine and the TIV vaccine 36 weeks later gives a better immune response than getting a placebo injection and the TIV vaccine 36 weeks later.

DNA Vaccine: Most vaccines are made of proteins and injected into a muscle. Proteins are natural substances that the body uses as building blocks. DNA serves as nature's code (instructions) for protein production in the body. A new kind of experimental vaccine being tested in this study is made from the DNA that is the code for three influenza proteins. It is a sterile preparation in a salt water solution. In this study, the DNA will be injected into a muscle. It will instruct the body to make a small amount of the influenza proteins.

Placebo Injection: The placebo in this study is a sterile salt water solution made for injection into people. It has no vaccine in it.

TIV: The vaccine injected at study week 36 will be a licensed seasonal trivalent influenza vaccine (TIV). It is not an experimental vaccine. It is made from influenza virus grown in hens' eggs, which is then inactivated, purified and prepared in a sterile preparation in a salt water solution.

STUDY PROCEDURES

Two groups of adults will be enrolled in the study. If you agree to take part in the study, you will be randomly assigned (by chance, like flipping a coin) to one of two injection schedules. All injections will be given in the upper arm muscle. Neither you nor the clinic staff will be informed about what schedule you are receiving until after everyone has completed all study injections. You will receive two injections during the study. Half the people in each age group will get the investigational DNA vaccine and the other half will get a placebo injection on the day of enrollment. Everyone in the study is scheduled to get the licensed TIV flu vaccine at Study Week 36 (this will be next flu season about September or October 2012).

DNA vaccine injections and placebo injections will be given using a needleless system called the Biojector 2000®. This device delivers the vaccine through the skin without the use of a needle. It uses the pressure of carbon dioxide instead of a needle to inject the vaccine through your skin and into the muscle. This system has FDA clearance for delivering vaccine injections into muscles. The TIV flu vaccine injections will be given using a needle and syringe.

You will have about 7 planned clinic visits and 2 telephone contacts during this study. Studies of investigational vaccines require following a set schedule for injections and follow-up visits in order to answer the study research questions. Some flexibility in scheduling is permitted, but it is important that you work with the staff to stay on schedule. The clinic staff will observe you for 15-30 minutes after each vaccination. One to two days after each injection, you must telephone the clinic staff to report on how you are doing. You will be asked to complete a diary card for 7 days at home. This will require that you record your temperature and symptoms and look at the injection site on your arm each day. The clinic staff is available to you by phone 24 hours a day to report any unexpected side effects.

You may record your symptoms on a paper diary card or enter them into a secure electronic form using the internet. If you choose to report your symptoms through the internet you will be trained by the clinic staff and given a username and password. If you have any symptoms that interfere with your usual activities, it may be necessary to come to the study clinic for an examination before your next scheduled visit. It is very important that you follow the instructions given to you by the clinic staff.

At each visit, you will be checked for any health changes or problems since your last visit. You will be asked how you are feeling and if you have taken any medications. Blood will be drawn at scheduled study visits to check on your health and to study your immune response to the vaccine. You will be told promptly if any of your test results show a health problem. Results of immune response tests are not tests used to check on your health and will not be given to you during the study. The study schedule is shown in the following table:

**Week of Study**	Start	Week 1	Week 4	Week 24	Week 36	Week 37	Week 40	Week 60
Informed Consent	X							
Health review and check-up	X		X	X	X		X	X
Study Injection	X				X			
Begin 7-Day Diary Card	X				X			
Telephone contact		X				X		
Pregnancy test (if applicable)	X		X		X			
Blood drawn; including samples stored for research	X		X	X	X		X	X

The amount of blood drawn will vary from about 3 tablespoons (45 mL) to about 7 tablespoons (105 mL), depending on the visit. You might also be asked to have laboratory tests between regular visits if needed to evaluate a change in your health.

MONITORING OF THE STUDY

This study will be monitored by a group of physicians and scientists associated with the National Institutes of Health. This group will review the information from the study and will pay close attention to harmful reactions.

GENETIC TESTING

Some of the blood drawn from you as part of this study will be used for genetic tests. There are many different types of genetic tests. In vaccine research, some genetic tests are done to see if different types of immune responses to a vaccine seem to be related to genetic differences in people. These tests will be done in a research lab using your stored samples. Genetic tests done in a research lab will **not** be in your medical record. Tests that are done in a research lab will **not** have your name on the sample given to the research lab. In the future, genetic research tests to help understand how vaccines work may be done on your DNA using stored samples.

STORED SAMPLES

You may not participate in this study if you are not willing to have your blood samples stored for future research purposes.

During your participation on this study blood samples will be collected from you. We will store these samples for future research to learn more about influenza virus, vaccines, the immune system, and/or other medical conditions.

The results from the research done with your stored samples will not be given to your health care provider and will not be put in your medical record. This is because the research test results, unlike routine medical testing, will not be for evaluating your health.

Labeling of Stored Samples

Your stored samples will be labeled by a code (such as a number) that only the study team can link to you. Any identifying information about you will be kept confidential to the extent permitted by law. Despite protections, there is a small chance that information identifying you will be given to someone who should not get it.

Future Studies

In the future, other investigators at NIH or outside of NIH may wish to study your stored samples. When the study team shares your stored samples, they will be marked with a code, but will not have any identifying information on them. Some information about you, such as your gender, age, health history, or ethnicity may also be shared with other investigators. Any future research studies using your samples will be reviewed by the investigator’s Institutional Review Board (IRB), a special committee that oversees medical research studies to protect the rights and welfare of human subjects.

Your stored materials will be used only for research and will not be sold. The research done with your materials may be used to develop new products in the future but you will not receive payment for such products.

POSSIBLE STUDY RISKS

Possible **risks from the injections:** temporary stinging, pain, redness, soreness, itchiness, swelling, bruising, or a cut in the arm. There is a very small chance of infection.

Possible **risks of blood drawing:** pain, bleeding, bruising, feeling lightheaded, or fainting.

Possible **risks from genetic testing:** unintended release of information that could be used by insurers or employers; discovering a gene or HLA type that suggests risk of disease for you or your family; discovering undisclosed family relationships.

Possible **risks from any vaccine:** fever, chills, rash, aches and pains, nausea, headache, dizziness, and fatigue. Some people have allergic reactions to vaccines. These types of reactions are usually greatest within the first 24 hours after vaccination and typically last 1 to 3 days. Over-the-counter medicine, such as acetaminophen, will generally help relieve symptoms from vaccination and may be used.

Possible **risks from DNA vaccine:** temporary drop in white blood cell count, sore arm, skin rash or hives. In theory, DNA vaccines could cause muscle damage, antibodies to DNA causing illness, or could get into the body's DNA leading to cancer or into the DNA of a bacteria or virus in your body. None of these possible risks have been seen in laboratory tests or so far in animals or humans who have taken DNA vaccines. Similar experimental DNA vaccines against a variety of infections have been given to about 2000 people.

Some people who have received a DNA vaccine through the Biojector® had a small red bump and then a scab where the shot was given.

Possible **risks from the seasonal influenza TIV:** Some people have fever, muscle and general body aches, headache, fatigue, and nausea. A severe allergic reaction can occur especially in people that are allergic to eggs. In 1976, a small number of people who got an inactivated swine flu vaccine developed a severe nerve weakness called Guillain-Barré syndrome. Guillain-Barré has not been linked to any subsequent influenza vaccines.

There may be side effects from either of the study vaccines- even serious or life threatening ones- that we do not yet know about. Please tell the study staff about any side effect you think you are having. This is important for your safety.

Possible **risks from Pregnancy:** We do not know the effects of the DNA vaccine on a fetus or nursing infant. Therefore, women who can have children must not breast-feed and must use effective birth control starting at least 21 days before getting the first vaccine until 12 weeks after the first study injection. Effective birth control includes not having sex, or using condoms, a diaphragm or cervical cap with a spermicidal gel or foam, an intrauterine device, a male partner who had a vasectomy or birth control pills, patches or other prescription methods.

Pregnant women can receive the seasonal influenza TIV, which is the type of injection given at study week 36. We will ask about the outcome of any pregnancy that begins during study participation.

Other Risks: The safety of the vaccines in this study is unknown. It is unknown if the study vaccine may alter your response to any future infections you may have with influenza viruses. You will be made aware of significant health effects of the vaccine and serious side effects if they would occur in other subjects, and will be updated during the trial as needed.

You may not donate blood at a blood bank while participating in an investigational vaccine study for one year after the date of the last injection of an investigational vaccine.

POSSIBLE BENEFITS

This study is not designed to benefit you. Receiving the seasonal influenza TIV may protect you against influenza illness, but the TIV vaccine does not always work to prevent influenza. No one knows if the investigational vaccine works to prevent influenza or if it will change the effect of the TIV vaccine. You and others may benefit in the future from the information that will be learned from the study.

COSTS OF PARTICIPATION

There are no costs to you for participating in this study. All medical costs for care you receive outside this study will be paid by you or your health insurance carrier (if you have insurance). It is possible that you may have some expenses that are not covered by the study compensation provided.

COMPENSATION TO YOU FOR YOUR PARTICIPATION

You will be compensated [insert site IRB-approved amount] for each visit that does not include an injection but does include a blood draw and [insert] for each injection visit. For visits that do not include an injection or a blood draw, you will be compensated [insert]. The approximate total compensation is from [insert]. This will be based on the number of study visits you attend and study injections you receive. You will be paid throughout the study after each visit.

REASONS FOR REMOVING YOU FROM THE STUDY WITHOUT YOUR CONSENT

The study doctor can take you out of this study without your permission *if:*

- continuing in the study could harm you,
- you do not follow study instructions or keep appointments, or
- the study is stopped by the NIH, regulatory boards or the FDA.

If you agree to take part in this study, it is important for you to keep all your appointments. However, if you don't want to stay in the study, you can leave at any time. You will not lose any benefits that you would have had if you had not joined the study.

If you receive the first study injection but not the TIV for any reason, you will be asked to continue with follow-up visits until the end of the study. It is important to continue to monitor your health even if you do not receive the TIV at Week 36.

ALTERNATIVES

This study is not designed to treat any disease. You may choose to not participate.

CONFIDENTIALITY

When results of an NIH-supported research study are reported in medical journals, on internet at http://www.ClinicalTrials.gov, or at scientific meetings, the people who take part are not named and identified. In most cases, the NIH will not release any information about your research involvement without your written permission. However, if you sign a release of information form, for example, for an insurance company, the insurance company receives information from your medical record. This information might affect (either favorably or unfavorably) the willingness of the insurance company to sell you insurance.

The Federal Privacy Act protects the confidentiality of your medical records. However, you should know that the Act allows release of some information from your medical record without your permission, for example, if it is required by regulatory agencies that oversee the study, law enforcement officials, or other authorized people.

POLICY REGARDING RESEARCH-RELATED INJURIES

The study site will provide immediate medical care for any injury resulting from your participation in research here. In general, no long-term medical care or financial compensation for research-related injuries will be provided by the National Institutes of Health or the Federal Government. However, you have the right to pursue legal remedy if you believe that your injury justifies such action.

Problems or Questions. If you have any problems or questions about this study, or about your rights as a research participant, or about any research-related injury, contact the investigator of record,_______________. Others you may call are the Study Coordinator _____________at________. You may also call the __________ Patient Representative at ______________.

Please keep a copy of this document in case you want to read it again.

Adult Participant Consent:

I have read the explanation about this study and have been given the opportunity to discuss it and ask questions. I consent to take part in this study.

Participant Name (print)	Date and Time
Signature	

Investigator name (print)	Date
Signature	
Witness name (print)	Date
Signature	

Appendix II: Contact Information

Contact Information redacted

Contact Information redacted

Appendix III: Schedule of Evaluations

		Screen	VRC Schedule of Evaluations							
**Visit**		01	02	02A	03	04	05	05A	06	07
**Week of Study**		-10 to 0	W 0	W1	W 4	W 24	W 36	W 37	W 40	W 60
[1]**Day of Study**		-70 to 0	D 0	D7	D 28	D 168	D 252	D259	D 280	D 420
**Clinical Evaluations**	**Tube**									
[1]Informed Consent		X	X							
Physical exam for eligibility at screen; BP, pulse, temp, wt other visits; targeted exam.		X	X		X	X	X		X	X
Medical history targeted to eligibility at screen; interim history for AEs other visits		X	X		X	X	X		X	X
[2] Study Vaccinations			X				TIV			
Begin 7-Day Diary Card			X				X			
Telephone contact; clinic visit if indicated				X				X		
Counseling on pregnancy prevention			X		X					
CBC, platelets	EDTA	3	3		3					
[3] Pregnancy test: urine (or serum)		X	X		X		X			
Creatinine and ALT	SST	4								
**Research Immunology**										
Antibody assays and serum storage	SST	[16][4]	24		32	32	32		32	32
PBMC and plasma for storage	EDTA	[40][4]	80		60		60		60	60
**Daily Volume (mL)**		**63**	**107**	**-**	**95**	**32**	**92**	**-**	**92**	**92**
**Max. Cumulative Volume (mL)**		**63**	**170**	**-**	**265**	**297**	**389**	**-**	**481**	**573**

[1] Screening informed consent may be signed more than 8 weeks prior to study enrollment; screening evaluations may be repeated, if needed.
The screening blood draw for research does not need to be repeated if it was collected more than 70 days prior to enrollment.
Day 0=day of enrollment and (blinded) DNA or PBS vaccine injection. Day 0 evaluations prior to first injection are the baseline for assessing adverse events subsequently.
[2] Complete post vaccination evaluations (BP, pulse and injection site assessment) at 15-60 minutes after each study injection.
[3] Negative pregnancy test results must be confirmed for women of reproductive potential prior to administering the DNA vaccine injection; licensed TIV administration is open label. It is not contraindicated for pregnant women, but pregnancy testing will be conducted and the women informed of result prior to administering TIV as part of this research protocol. If pregnant, research blood draws will be one-fourth of shown volume or eliminated if the pregnant participant is anemic.
Schedule Visits 02A through 05 with respect to Day 0; the following Visit windows apply: 02A and 05A (+2 days); Visit 03 and 04 (±7 days); Visit 05 (±28 days). Schedule Visits 06 and 07 to be 4 and 24 weeks, respectively, after Visit 05; Visits 06 and 07 each have a ±7 day window.
[4]Storage samples of peripheral blood mononuclear cells (PBMCs) and serum collected during screening may be used for assay validation and site proficiency testing.

Appendix IV: Assessment of Relationship to Vaccine and Adverse Event Severity Grading

Assessment of Causality Relationship of an Adverse Event (AE) to Study Vaccine:

The relationship between an AE and the vaccine will be assessed by the investigator on the basis of his or her clinical judgment and the definitions below.

- **Definitely Related**. The AE and administration of study agent are related in time, and a direct association can be demonstrated.
- **Probably Related**. The AE and administration of study agent are reasonably related in time, and the AE is more likely explained by study agent than other causes.
- **Possibly Related**. The AE and administration of study agent are reasonably related in time, but the AE can be explained equally well by causes other than study agent.
- **Not Related**. There is not a reasonable possibility that the AE is related to the study agent.

For purposes of preparing data reports in which AE attributions are limited to "**Related**" or "**Not Related**", in this protocol, the "Definitely, Probably and Possibly" attributions will be mapped to the "Related" category. The definitions that apply when these two categories alone are used are as follows:

- **Related** – There is a reasonable possibility that the AE may be related to the study agent.
- **Not Related** – There is not a reasonable possibility that the AE is related to the study agent.

Grading the Severity of Adverse Events:
The FDA Guidance for Industry (September 2007): "Toxicity Grading Scale for Healthy Adult and Adolescent Volunteers Enrolled in Preventive Vaccine Clinical Trials" is the basis for the severity grading of adverse events in this protocol. Several modifications were made to the table as follows:

- "Emergency room visit" is not automatically considered a life-threatening event; these words have been removed from any "grade 4' definition where they appear in the table copied from the guidance document.
- Any laboratory value shown as a "graded" value in the table that is within the institutional normal range will not be severity graded or recorded as an adverse event.
- Severity grading for hemoglobin decrease on the basis of the magnitude of decrease from baseline is not applicable at the grade 1 level; only absolute hemoglobin will be used to define grade 1 decrease. Increases in hemoglobin are AEs only for values above the upper limit of normal and are graded by the systemic illness clinical criteria.
- Severity grading definition for Grade 4 local reaction to injectable product (Erythema/Redness and Induration/Swelling) included added text "requiring medical attention".
- 1 X ULN was removed from the definition for PT increase.

When not otherwise specified in the table, the following guidance will be used to assign a severity grade:

Grade 1 (Mild): No effect on activities of daily living

Grade 2 (Moderate): Some interference with activity not requiring medical intervention

Grade 3 (Severe): Prevents daily activity and requires medical intervention

Grade 4 (Life-threatening): Hospitalization; immediate medical intervention or therapy

required to prevent death.

Grade 5 (Death): Death is assigned a Grade 5 severity.

Only the single adverse event that is assessed as the primary cause of death should be assigned "grade 5" severity.

Toxicity Grading Scale for Healthy Adult and Adolescent Volunteers Enrolled in Preventive Vaccine Clinical Trials
FDA Guidance - September 2007

A. Tables for Clinical Abnormalities

Local Reaction to Injectable Product	Mild (Grade 1)	Moderate (Grade 2)	Severe (Grade 3)	Potentially Life Threatening (Grade 4)
Pain	Does not interfere with activity	Repeated use of non-narcotic pain reliever > 24 hours or interferes with activity	Any use of narcotic pain reliever or prevents daily activity	Hospitalization
Tenderness	Mild discomfort to touch	Discomfort with movement	Significant discomfort at rest	Hospitalization
[1] Erythema/Redness	2.5 – 5 cm	5.1 – 10 cm	> 10 cm	Necrosis or exfoliative dermatitis requiring medical attention
[2] Induration/Swelling	2.5 – 5 cm and does not interfere with activity	5.1 – 10 cm or interferes with activity	> 10 cm or prevents daily activity	Necrosis requiring medical attention
**[3] Vital Signs**	**Mild (Grade 1)**	**Moderate (Grade 2)**	**Severe (Grade 3)**	**Potentially Life Threatening (Grade 4)**
[4] Fever (°C)<br>(°F)	38.0 – 38.4<br>100.4 – 101.1	38.5 – 38.9<br>101.2 – 102.0	39.0 – 40<br>102.1 – 104	> 40<br>> 104
Tachycardia - beats per minute	101 – 115	116 – 130	> 130	Hospitalization for arrhythmia
[5] Bradycardia - beats per Minute	50 – 54	45 – 49	< 45	Hospitalization for arrhythmia
Hypertension (systolic) - mm Hg	141 – 150	151 – 155	> 155	Hospitalization for malignant hypertension
Hypertension (diastolic) - mm Hg	91 – 95	96 – 100	> 100	Hospitalization for malignant hypertension
Hypotension (systolic) – mm Hg	85 – 89	80 – 84	< 80	Hospitalization for hypotensive shock
Respiratory Rate – breaths per minute	17 – 20	21 – 25	> 25	Intubation

1. In addition to grading the measured local reaction at the greatest single diameter, the measurement should be recorded as a continuous variable.
2. Induration/Swelling should be evaluated and graded using the functional scale as well as the actual measurement.
3. Subject should be at rest for all vital sign measurements.
4. Oral temperature; no recent hot or cold beverages or smoking.
5. When resting heart rate is between 60 – 100 beats per minute. Use clinical judgment when characterizing Bradycardia among some healthy subject populations, for example, conditioned athletes.

Systemic (General)	Mild (Grade 1)	Moderate (Grade 2)	Severe (Grade 3)	Potentially Life Threatening (Grade 4)
Nausea/vomiting	No interference with activity or 1 – 2 episodes/24 hours	Some interference with activity or > 2 episodes/24 hours	Prevents daily activity, requires outpatient IV hydration	Hospitalization for hypotensive shock
Diarrhea	2 – 3 loose stools or < 400 gms/24 hours	4 – 5 stools or 400 – 800 gms/24 hours	6 or more watery stools or > 800gms/24 hours or requires outpatient IV hydration	Hospitalization
Headache	No interference with activity	Repeated use of non-narcotic pain reliever > 24 hours or some interference with activity	Significant; any use of narcotic pain reliever or prevents daily activity	Hospitalization
Fatigue	No interference with activity	Some interference with activity	Significant; prevents daily activity	Hospitalization
Myalgia	No interference with activity	Some interference with activity	Significant; prevents daily activity	Hospitalization

Systemic Illness	Mild (Grade 1)	Moderate (Grade 2)	Severe (Grade 3)	Potentially Life Threatening (Grade 4)
Illness or clinical adverse event (as defined according to applicable regulations)	No interference with activity	Some interference with activity not requiring medical intervention	Prevents daily activity and requires medical intervention	Hospitalization

B. Tables for Laboratory Abnormalities

The laboratory values provided in the tables below serve as guidelines and are dependent upon institutional normal parameters. Institutional normal reference ranges should be provided to demonstrate that they are appropriate.

Serum *	Mild (Grade 1)	Moderate (Grade 2)	Severe (Grade 3)	Potentially Life Threatening (Grade 4)**
Sodium – Hyponatremia mEq/L	132 – 134	130 – 131	125 – 129	< 125
Sodium – Hypernatremia mEq/L	144 – 145	146 – 147	148 – 150	> 150
Potassium – Hyperkalemia mEq/L	5.1 – 5.2	5.3 – 5.4	5.5 – 5.6	> 5.6

**Serum ***	**Mild<br>(Grade 1)**	**Moderate<br>(Grade 2)**	**Severe<br>(Grade 3)**	**Potentially Life Threatening<br>(Grade 4)****
Potassium – Hypokalemia mEq/L	3.5 – 3.6	3.3 – 3.4	3.1 – 3.2	< 3.1
Glucose – Hypoglycemia mg/dL	65 – 69	55 – 64	45 – 54	< 45
Glucose – Hyperglycemia<br>Fasting – mg/dL<br>Random – mg/dL	100 – 110<br>110 – 125	111 – 125<br>126 – 200	>125<br>>200	Insulin requirements or hyperosmolar coma
Blood Urea Nitrogen<br>BUN mg/dL	23 – 26	27 – 31	> 31	Requires dialysis
Creatinine – mg/dL	1.5 – 1.7	1.8 – 2.0	2.1 – 2.5	> 2.5 or requires dialysis
Calcium – hypocalcemia mg/dL	8.0 – 8.4	7.5 – 7.9	7.0 – 7.4	< 7.0
Calcium – hypercalcemia mg/dL	10.5 – 11.0	11.1 – 11.5	11.6 – 12.0	> 12.0
Magnesium – hypomagnesemia mg/dL	1.3 – 1.5	1.1 – 1.2	0.9 – 1.0	< 0.9
Phosphorous – hypophosphatemia mg/dL	2.3 – 2.5	2.0 – 2.2	1.6 – 1.9	< 1.6
CPK – mg/dL	1.25 – 1.5 x ULN***	1.6 – 3.0 x ULN	3.1 –10 x ULN	> 10 x ULN
Albumin – Hypoalbuminemia g/dL	2.8 – 3.1	2.5 – 2.7	< 2.5	--
Total Protein – Hypoproteinemia g/dL	5.5 – 6.0	5.0 – 5.4	< 5.0	--
Alkaline phosphate – increase by factor	1.1 – 2.0 x ULN	2.1 – 3.0 x ULN	□3.1– 10 x ULN	> 10 x ULN
Liver Function Tests – ALT, AST<br>increase by factor	1.1 – 2.5 x ULN	2.6 – 5.0 x ULN	5.1 – 10 x ULN	> 10 x ULN
Bilirubin – when accompanied<br>by any increase in Liver Function Test<br>increase by factor	1.1 – 1.25 x ULN	1.26 – 1.5 x ULN	1.51 – 1.75 x ULN	> 1.75 x ULN
Bilirubin – when Liver Function Test is normal; increase by factor	1.1 – 1.5 x ULN	1.6 – 2.0 x ULN	2.0 – 3.0 x ULN	> 3.0 x ULN
Cholesterol	201 – 210	211 – 225	> 226	---
Pancreatic enzymes – amylase, lipase	1.1 – 1.5 x ULN	1.6 – 2.0 x ULN	2.1 – 5.0 x ULN	> 5.0 x ULN

* The laboratory values provided in the tables serve as guidelines and are dependent upon institutional normal parameters. Institutional normal reference ranges should be provided to demonstrate that they are appropriate.
** The clinical signs or symptoms associated with laboratory abnormalities might result in characterization of the laboratory abnormalities as Potentially Life Threatening (Grade 4). For example, a low sodium value that falls within a grade 3 parameter (125-129 mE/L) should be recorded as a grade 4 hyponatremia event if the subject had a new seizure associated with the low sodium value.
***ULN" is the upper limit of the normal range.

**Hematology ***	**Mild (Grade 1)**	**Moderate (Grade 2)**	**Severe (Grade 3)**	**Potentially Life Threatening (Grade 4)**
Hemoglobin (Female) - gm/dL	11.0 – 12.0	9.5 – 10.9	8.0 – 9.4	< 8.0
Hemoglobin (Female) decrease from baseline value - gm/dL	not applicable	1.6 – 2.0	2.1 – 5.0	> 5.0
Hemoglobin (Male) - gm/dL	12.5 – 13.5	10.5 – 12.4	8.5 – 10.4	< 8.5
Hemoglobin (Male) decrease from baseline value – gm/dL	not applicable	1.6 – 2.0	2.1 – 5.0	> 5.0
WBC Increase - cell/mm^3	10,800 – 15,000	15,001 – 20,000	20,001 – 25, 000	> 25,000
WBC Decrease - cell/mm^3	2,500 – 3,500	1,500 – 2,499	1,000 – 1,499	< 1,000
Lymphocytes Decrease - cell/mm^3	750 – 1,000	500 – 749	250 – 499	< 250
Neutrophils Decrease - cell/mm^3	1,500 – 2,000	1,000 – 1,499	500 – 999	< 500
Eosinophils - cell/mm^3	650 – 1500	1501 - 5000	> 5000	Hypereosinophilic
Platelets Decreased - cell/mm^3	125,000 – 140,000	100,000 – 124,000	25,000 – 99,000	< 25,000
PT – increase by factor (prothrombin time)	1.10 x ULN**	1.11 – 1.20 x ULN	1.21 – 1.25 x ULN	> 1.25 ULN
PTT – increase by factor (partial thromboplastin time)	1.10 – 1.20 x ULN	1.21 – 1.4 x ULN	1.41 – 1.5 x ULN	> 1.5 x ULN
Fibrinogen increase - mg/dL	400 – 500	501 – 600	> 600	--
Fibrinogen decrease - mg/dL	150 – 200	125 – 149	100 – 124	< 100 or associated with gross bleeding or disseminated intravascular coagulation (DIC)

* The laboratory values provided in the tables serve as guidelines and are dependent upon institutional normal parameters. Institutional normal reference ranges should be provided to demonstrate that they are appropriate.
